# A scalable and efficient UAV-based pipeline and deep learning framework for phenotyping sorghum panicle morphology from point clouds

**DOI:** 10.1016/j.plaphe.2025.100050

**Published:** 2025-05-19

**Authors:** Chrisbin James, Shekhar S. Chandra, Scott C. Chapman

**Affiliations:** a School of Agriculture and Food Sustainability, The University of Queensland, Brisbane, Australia; b School of Electrical Engineering and Computer Science, The University of Queensland, Brisbane, Australia

**Keywords:** Deep learning, Pointclouds, 3D object detection, High-throughput phenotyping, Sorghum

## Abstract

Sorghum canopy architecture in field trials is determined by various phenotypic traits, such plant and panicle count, leaf density and angle and panicle morphology, and canopy height. These traits together affect light capture and biomass production as well as conversion of photosynthates to grain yield. Panicle morphology exhibits considerable variation as influenced by genetics, environmental conditions and management practices. This study presents a framework for the 3D reconstruction of sorghum canopies and phenotyping panicle morphology. First, we developed a scalable, low-altitude Unmanned Aerial Vehicle (UAV)-based protocol that leverages videos for efficient data acquisition, combined with Neural Radiance Fields (NeRF)s to generate high-quality 3D point cloud reconstructions of sorghum canopies. Next, a 3D model was built to simulate 3D sorghum canopies to create annotated datasets for training deep learning-based semantic segmentation and panicle detection algorithms. Finally, we propose SegVoteNet, a novel multi-task deep learning model that integrates VoteNet and PointNet++ within a shared backbone architecture. Designed for semantic segmentation and 3D detection on pure point cloud data, SegVoteNet incorporates a voting and sampling module that leverages segmentation results to optimize object proposal generation. SegVoteNet is robust, achieving 0.986 Mean Average Precision (mAP) @ 0.5 Intersection Over Union (IOU) on synthetic datasets, and 0.850 mAP @ 0.5 IOU on real point cloud datasets for sorghum panicle detection, without fine-tuning. This set of pipelines provides a robust scalable method for phenotyping sorghum panicles in field trials in breeding and commercial applications. Further work is developing a capability to estimate grain number per panicle, which would provide breeders with additional phenotypes to select.

## 1 Introduction

Sorghum (Sorghum bicolor) breeders develop varieties that must withstand hostile (hot, droughted) environments in which the maintenance of grain yield, and specifically grain number per unit area, is a key characteristic. Although the total mass of grain is the main trait of interest, there is little documentation of how grain number is directly affected by the physical phenotypes that determine the overall canopy structure of sorghum in field trials. These may include panicle and tiller count, panicle morphology (branching and grain numbers per panicle) and canopy height. Compared to other cereal crops, sorghum displays substantial variations in panicle shape and structure impacted by genetics and the growing environment. The shape of the sorghum panicle is influenced by the number and length of its branches. Additionally, the preferred panicle architecture depends on environmental conditions—such as hot and dry versus wet and cold climates—as well as the intended purpose of cultivation, whether for grain yield or biomass production [1]. Panicle count and tillering are key determinants of both biomass and grain yield (via grain number per unit area) while also shaping canopy architecture by affecting traits like density and leaf angle [2]. Beyond its role in grain yield, panicle morphology differs between wild and domesticated sorghum varieties, with wild sorghum typically exhibiting more open and loosely structured panicles [3]. A study by Sharma et al. found that panicle compactness is significantly correlated with grain mould infestation, as compact panicles tend to retain more moisture, increasing the risk of mould development. Moreover, compact panicles have been linked to higher incidences of head bugs [4] and webworms [5]. While compact panicle varieties are more common in hot, dry regions, looser panicle varieties are better suited to wet and colder climates [6].Phenotyping panicle morphology is essential to understanding its impact on grain yield, pest and disease resistance, and adaptation to diverse environmental conditions.

Various machine learning and deep learning algorithms have been applied to estimate panicle (or head) numbers from image data in several grain crop species. These algorithms typically leverage Red Green Blue (RGB) imagery collected from field cameras, smartphones, and/or UAVs. The availability of publicly accessible datasets, such as the Global Wheat Head Detection Dataset (GWHD) [7,8], the Maize Tassel Detection Dataset (MTC) [9], and the sorghum dataset [10], has facilitated the development of robust panicle identification models. Most of these models are either detection-based (single and two-stage), providing the position and an estimate of panicle dimensions via bounding boxes [11,12], or regression-based [[13], [14], [15]], which are lighter with fewer model parameters, offering faster training and inference times. Regression-based models estimate both the count and position of the panicles but do not account for the size of the panicles.

Although there has been extensive research into the application of 2D images for plant phenotyping, relatively few studies have focused on 3D modalities such as point clouds and meshes. However, 3D data provides a more detailed and comprehensive view of plants, making it particularly useful for phenotyping complex traits that are difficult to observe with 2D images alone. These traits include the morphology and shape of individual organs, plant height, leaf angle, plant volume, and stem diameter [16]. A major challenge in 3D plant phenotyping is the cost and scalability of data acquisition. For indoor environments, challenges like background noise, ambient lighting, and occlusion can be managed more effectively by controlling the phenotyping setup, leading to high-quality data with minimal noise. In contrast, field-based 3D phenotyping systems must address these challenges either through post-processing steps after data acquisition or by developing algorithms capable of handling noise and occlusion directly in the analysis. This makes data capture more complext in field-based phenotyping but essential for describing realistic plant responses under natural conditions. 3D phenotyping is particularly useful for tracking dynamic plant processes like canopy development, leaf expansion, and stem elongation over time, even at the individual plant or organ level. Applications include describing leaf characteristics, distinguishing between weeds and crops, estimating biomass, and classifying plant organs by size and shape [17].

## 2 Related work

### 2.1 Indoor phenotyping methods

For sorghum, various 3D indoor phenotyping methods using different sensors have been proposed, typically focusing on imaging a single plant at a time. Xiang et al. [18] utilized an Xbox Kinect V2 with a depth camera, RGB camera, and infrared emitter to image potted sorghum plants. The camera was mounted on a linear actuator and captured images at multiple heights, with approximately 90 ​% overlap between consecutive images. The RGB-D images were then co-registered to create a point cloud model of the plant, followed by denoising and skeletonization to segment the plant into stem and leaves. The point cloud-derived measurements of stem diameter and leaf angle showed strong correlations with the measured values, achieving an Mean Absolute Error (MAE) of 1.10 ​mm and 2.24°. Gaillard et al. [19] introduced a voxel carving algorithm to reconstruct a 3D voxel model of a sorghum plant using five equispaced images. The images were calibrated and pre-processed to calculate transformation matrices to define the camera parameters, and a Convolutional Neural Network (CNN)-based segmentation model was employed to mask the background. The voxel carving algorithm used the segmentation results and camera poses to construct a 3D voxel grid outlining the plant's photo hull. The voxel-derived plant volume and surface area had a strong correlation with measured values, with R-squared values of 0.839 and 0.946, respectively. Lastly, Patel et al. [20] used a Leica BLK360 high-resolution laser scanner to scan sorghum plants indoors. They evaluated multiple deep learning-based point cloud segmentation algorithms to segment the point cloud into stem, leaf, and diameter components, with PointNet++ [21] yielding the most robust results. Measurements of plant height, diameter, and compactness derived from the segmented point clouds showed a strong correlation with ground truth, with Root Mean Square Error (RMSE) values of 1.07 ​cm, 1.58 ​cm, and 0.03 ​cm, respectively.

### 2.2 Outdoor phenotyping methods

Outdoor 3D phenotyping methods have also been explored for phenotyping sorghum canopies in the field. Malambo et al. [22] utilized a high-density terrestrial LiDAR scanner (FARO Focus X330), mounted on a sprayer tractor at a height of 7 ​m. The LiDAR scanner collected RGB imagery concurrently with 3D point cloud data, allowing colour values to be interpolated and overlaid onto the scanned points. The point cloud data was then processed to segment and detect individual instances of panicles. Initially, a colour threshold was applied to filter out foliage, alongside the exclusion of low-altitude points. The filtered points were then grouped into clusters using the DBSCAN algorithm [23], based on the XY coordinates of the point cloud. Finally, the localised point cloud clusters were binned into 3 ​cm vertical slices to determine the individual profiles of the panicles. Plant height measurements from the LiDAR scanner showed a strong correlation with ground truth, with R values of 0.87 and RMSE of 11.4 ​cm. However, panicle-specific measurements (height and width) exhibited weaker precision, with correlation values of 0.79 for panicle height and 0.63 for panicle width, and RMSE values of 2.48 ​cm and 1.49 ​cm, respectively. The panicle detection accuracy was 89.3 ​%. More recently, Zarei et al. proposed PlantSegNet [24], a two-stage deep learning-based method for instance segmentation of plant organs, including leaves, stems, and panicles. They investigated the use of stereo laser scanners for acquiring high-resolution point clouds of sorghum plants under field conditions (focusing on a small area). Additionally, the authors explored the use of synthetic 3D sorghum models to generate annotated training data, aiming to reduce manual annotation efforts. The models were trained on synthetic datasets and evaluated on real annotated point clouds. Although there was some degradation in performance for the instance segmentation results, the models demonstrated a relatively strong ability to generalise to real point cloud models, achieving an Average Precision (AP) of 0.72 on the synthetic dataset and 0.69 on the real dataset. PlantSegNet employs an EdgeConv-based backbone network [25] for feature extraction. Furthermore, the authors proposed a piecewise loss function that imposes stricter penalties based on the similarity between the learnt features for points belonging to different instances, particularly those near the junction areas of stems and leaves. Laser scanners and LiDARs allow for capturing 3D point cloud data in outdoor environments, but the scalability and the speed of data acquisition can potentially be a limiting factor as the number of plots and the size of the area to be digitised increases, and the sensors have historically been much more expensive than image-based sensors). Especially with Laser Scanners, there is a tradeoff between capture of high-resolution 3D point cloud data, vs limited size of the region of interest, in addition to a need for the calibration and set up of the sensors to deal with outdoor lighting and operation under high temperature conditions.

### 2.3 NeRF-based 3D reconstruction

There has been a paradigm shift in RGB-image-based 3D reconstruction, with NeRF-based deep neural rendering methods [26] emerging as an alternative to traditional Structure from motion (Sfm) pipelines to generate high-quality point clouds. NeRFs learn a 3D scene representation as a mapping function using a neural network. For any given point in 3D space and its corresponding viewing direction, the network predicts the observed colour and density. Novel views and new images are generated by integrating the colour and density values along rays cast from the camera's position. For instance, Arshad et al. [27] assess various NeRF-based methods in both indoor and outdoor conditions in multiple scales for maize plant 3D reconstruction, via RGB videos (at 4K resolution) recorded from an iPhone 13 pro. The point cloud geometry generated by NeRF models was compared to reference point clouds obtained via co-registering multiple terrestrial LiDAR scans collected via a Faro Focus S350. Results showed that the NeRFacto model, developed by Tancik et al. bundled with the Nerfstudio framework [28], was the most robust across different environments for generating high-fidelity point clouds. It outperformed other models such as Instant-NGP [29] and TensorRF [30], making it ideal for tasks requiring very detailed point cloud models.

NeRFacto is an ensemble model that combines components from existing NeRF frameworks, optimised for real-world data capture. Key contributions include the pose refinement module, which corrects camera poses during training by backpropagating loss gradients, along with the proposal sampler module, which enhances the reconstruction quality via employing a fused-Multi Layer Perceptron (MLP) with hash encoding to sample from optimal regions across the scene while generating renders. NeRFacto achieved the highest precision, recall, and F1-score for point cloud models and the best image quality metrics (Peak Signal-to-Noise Ratio, Structural Similarity Index Measure, and Learned Perceptual Image Patch Similarity) for novel view renders. Ying et al. present a NeRF-based 3D reconstruction pipeline for in-situ phenotyping of pepper plants in a greenhouse setting. A GoPro Hero 11 camera, recording RGB videos in 4K resolution at 120 Frames per second (fps), is employed for NeRF-based reconstruction, while an RVC-X mini 3D laser scanner is used to collect reference point cloud models for validation. Both the camera and the laser scanner are mounted on a robotic arm programmed to capture images of the plants. Instant-NGP and Instant-NSR [31] NeRF methods are compared and evaluated for their effectiveness in 3D reconstruction. Following this, individual peppers are segmented from the 3D plant model by processing the point cloud through PointNet++ [21]. The MAE between the NeRF-derived and laser scanner reference point clouds for individual peppers was approximately 1 ​mm across all three axes. Ye et al. [32] present an optimised pipeline for 3D reconstruction of tomato fruits from in-situ multi-view image captures. The images are processed via a feature point extraction module that employs Scale invariant feature transform (SIFT) [33] and Fast Library for Approximate Nearest Neighbors (FLANN) [34] for feature extraction and matching. Followed by, Random Sample Consensus (RANSAC) [35] for eliminating mismatched feature points, and using Sfm to facilitate the generation of an initial sparse point cloud model. This Sfm-derived point cloud model is then used to initialise 3D Gaussian Splatting (3DGS) [36], which is used for texture rendering and deriving the point cloud model. Finally, Poisson surface reconstruction is applied to the point cloud to generate the final surface model.

### 2.4 UAV-based 3D reconstruction methods

Although the majority of the UAV-based phenotyping developed methods focus on 2D image-based data acquisition, there have been methods proposed in recent years that focus on employing Sfm-based photogrammetry for reconstructing 3D canopy architecture. Liu et al. [37] introduced a UAV-based protocol for 3D reconstruction of maize and soybean canopies in the field. The drone flies in a circular pattern above each plot with a 45-degree gimbal angle, aiming the camera toward the plot centre. At an altitude of 5 ​m and a flight speed of 0.2 ​m/s, the radius for each circle was 5 ​m, and the data acquisition time for each plot was around 5 ​min. After processing the UAV image sequence, the point cloud model was denoised and filtered to remove background points. A 3D model for the plants was obtained by triangulating the filtered point cloud. The derived leaf number, leaf area, and plant height measurements correlated well with ground truth for early growth stages. In maize, a 100 ​% reconstruction of leaf number was achieved at 43-Days after sowing (DAS), while 88.6 ​% reconstruction was achieved at 91-DAS. In soybean, 81 ​% and 90.2 ​% leaf reconstruction was observed at 43-DAS and 91-DAS, respectively. However, the total leaf area derived from the UAV models was notably smaller than the ground truth, with the percentage of leaf area missed by the models increasing with canopy development. For maize, the UAV-derived 3D model missed 21.7 ​% leaf area at 43-DAS and 41.88 ​% at 91-DAS. In soybean, 44.9 ​% and 63.5 ​% leaf area was missed at 43-DAS and 91-DAS, respectively. Xiao et al. [38] propose a Cross-Circular Oblique (CCO) flight route to improve canopy coverage and efficiency in image acquisition. The CCO technique captures images from multiple viewing angles around the plot, ensuring high image overlap and efficient image utilization compared to multidirectional oblique or traditional grid flight patterns. This flight plan was tested for maize, sugar beet, and cotton canopies at 47, 71, and 90 DAS. In maize, leaf length and width measurements derived from 3D models reconstructed using the CCO route correlated well with ground truth, with an R-squared value of 0.93 and an RMSE of 3.05 ​cm. UAV-based 3D reconstruction methods have also been tested in sorghum, specifically for detecting and identifying panicle geometry. Chang et al. [39] test a low-altitude 10m nadir UAV flight for reconstructing 3D point cloud models of sorghum canopies. A panicle detection algorithm was also proposed to process the point cloud models to identify individual panicles. Firstly, the point cloud model is filtered via a RGB-based threshold to extract points belonging to the panicles. The filtered points are denoised and processed by a disk-fitting algorithm based on elevation values to identify and localise a single panicle instance. The panicle detection algorithm was tested on point cloud models for 1m (sampled from the center of the plot) single row plots, for seventeen different varieties. The panicle detection algorithm was able to estimate panicle count with a R-squared value of 0.83. The panicle length and diameter values were estimated from the point cloud models correlated with the ground truth measurements with R-squared values of 0.38 and 0.69. However, the authors note a key limitation of the detection algorithm: it failed to identify small or emerging panicles and often detected closely clustered panicles as a single instance. This issue highlights the challenge of distinguishing individual panicles, especially when they are in close proximity with each other or are not fully developed. Beyond flight pattern optimization, recent studies have explored the use of NeRF-based methods as an alternative to traditional Sfm-based photogrammetry for processing UAV datasets [40,41]. These approaches primarily focus on improving data processing efficiency for large-scale survey reconstruction rather than canopy or plot-scale reconstruction for plant phenotyping applications.

### 2.5 Synthetic datasets

Functional-Structural Plant Models (Functional structural plant model (FSPM)s) have been widely used in various studies to generate annotated plant geometry models for training deep learning models [42]. Several image-based Simulated-to-Real (images) (Sim2Real) dataset generation approaches have leveraged FSPMs. For example, Li et al. introduced the D3P framework, which integrates a 3D wheat model with a ray tracer to generate realistic images with automatic leaf tip labels. This framework allows for the manipulation of factors such as canopy structure, leaf texture, soil properties, and lighting conditions to enhance realism. To mitigate the domain gap between simulated and real images, CycleGAN [43] is employed for domain adaptation, producing datasets for training detection models. Similarly, Helmrich et al. developed Synavis, an end-to-end framework that combines the CPlantBox FSPM [44] with Unreal Engine [45] to render plant structures at multiple scales. This enables real-time generation of annotated datasets for training deep learning models [46]. Beyond image-based approaches, FSPMs have also been applied to 3D point cloud datasets. For instance, Turgut et al. evaluated different deep learning architectures for semantic segmentation of point cloud models of rosebush plants. They simulated rosebush geometry using the procedural model proposed by Favre et al. [47] within the L-Studio software [48], generating annotated point clouds that segmented flowers, leaves, and stems [49]. Another example is the Arabidopsis segmentation model, Stratified Plant Transformer, developed by Zheng et al., which was trained on an L-System-based structural plant model to generate annotated point cloud datasets for segmenting leaves, stems, and flowers [50]. However, there are currently no publicly available FSPM models capable of simulating full-scale sorghum canopies with accurate and diverse panicle architectures. Zarei et al. recently introduced a procedural framework for high-resolution sorghum simulation to generate annotated point clouds for instance segmentation tasks [24], leveraging the 3D sorghum plant dataset from Gaillard et al. [19] as a foundation for plant geometries.

### 2.6 Plot scale 3D sorghum panicle detection and segmentation

While Zarei et al.’s PlantSegNet demonstrates accurate instance segmentation for sorghum in field conditions, it was not specifically designed for panicle counting and is limited to single plant instances. Furthermore, instance segmentation may be unreliable for dense or open panicle structures, where separating and assigning points to individual panicles is not feasible. The gantry-based data acquisition method used in this approach is also relatively slow and challenging to scale for large, experiments with dense canopies. In contrast, Malambo et al. and Chang et al. propose more high-throughput data acquisition methods. However, their panicle detection approaches, which rely on shape fitting and density-based clustering, struggle to distinguish closely spaced panicles, partly due to lower-resolution 3D reconstructions and limited method reliability for more diverse panicle structures.

In this paper, we introduce a framework (based on open source and commercial software/libraries for photogrammetry, image processing and deep-learning) for the reconstruction and processing of 3D point cloud models of sorghum canopies in field settings, with a focus on phenotyping panicle geometry. Our approach is summarised as follows:

• A scalable low-altitude UAV-based pipeline for high-quality 3D reconstruction of sorghum canopies, leveraging RGB video for rapid data acquisition. The pipeline integrates optimised flight patterns, automated Ground Control Point (GCP) detection using occlusion-tolerant STag markers [51], and NeRF to enhance scene reconstruction.
• A procedural 3D sorghum canopy generation model developed from high-quality reference 3D sorghum panicle models, along with synthetic annotated point cloud datasets for semantic and instance segmentation (for panicle, stem, and leaf) and 3D detection of sorghum panicles.
• SegVoteNet: A multi-task deep learning model specifically designed for semantic segmentation and 3D object detection of pure point cloud models of sorghum canopies. Featuring a voting and sampling module optimised for 3D panicle detection that uses segmentation labels for seed point sampling to generate object proposals.

## 3 Materials and methods

Our framework is and end-to-end methodology, beginning with UAV data acquisition and image processing for point cloud reconstruction, followed by the analysis of point cloud models for panicle detection.

### 3.1 3D reconstruction pipeline for sorghum canopies in the field

#### 3.1.1 Trial design and layout

A sorghum trial was planted at the University of Queensland's Gilbert site, located on the Gatton campus (Latitude: 27.5589, Longitude:152.332923), on October 23, 2023, as part of Australia's Grains Research and Development Corporation (GRDC) Innovations in National Variety Testing (INVITA) project [52]. The trial consisted of 14 commercial grain sorghum cultivars, with two treatments: rainfed and irrigated, each replicated twice. Table 1 provides an overview of the trial layout, detailing the allocation of cultivars to the plots.

**Table 1:** Gilbert sorghum trial design.

Range/Row	Cultivar			Treatment-Replication
	Row 1	Row 2	Row 3	
**Range 1**	Acclaim	MR-Bazley 2	Resolute	Irrigated 1
**Range 2**	Halifax	Sentinel-IG	MR-Taurus	
**Range 3**	Liberty	A66	A14	
**Range 4**	Cracker	MR-Buster	Filler	
**Range 5**	A88	Viper-IG	G33	
**Range 6**	MR-Bazley	Liberty	G33	Irrigated 2
**Range 7**	A14	Acclaim	MR-Buster	
**Range 8**	Cracka	Viper-IG	MR-Taurus	
**Range 9**	A88	Sentinel-IG	A66	
**Range 10**	Resolute	Halifax	Filler	
**Range 11**	Buffer	Buffer	Buffer	Buffer
**Range 12**	Acclaim	MR-Bazley	Resolute	Rainfed 1
**Range 13**	Halifax	Sentinel-IG	MR-Taurus	
**Range 14**	Liberty	A66	A14	
**Range 15**	Cracka	MR-Buster	Filler	
**Range 16**	A88	Viper-IG	G33	
**Range 17**	MR-Bazley	Liberty	G33	Rainfed 2
**Range 18**	A14	Acclaim	MR-Buster	
**Range 19**	Cracka	Viper-IG	MR-Taurus	
**Range 20**	A88	Sentinel-IG	A66	
**Range 21**	Resolute	Halifax	Filler	

Within each replicate, 15 plots are arranged in a 3 ​× ​5 grid, comprising 14 cultivars and one filler plot. Each plot, identified by row-range numbers, measures 5m ​× ​5.5m. The planting configuration for each plot includes 7 planted rows, with a plant spacing of ca 0.1m within each row and a row spacing of 0.75m.

#### 3.1.2 UAV campaign and flight plan

We used DJI Mavic 3 Enterprise (3E) Drone for experiments. The Mavic 3E is equipped with a 20-megapixel wide camera lens featuring a 24 ​mm format equivalent focal length and an adjustable aperture range from f/2.8 to f/11, and 84**^◦^** field of view. Previous studies on UAV-Sfm-based 3D canopy reconstruction primarily employ two variations of low-altitude flight plans, CCO and grid pattern flights, flying at approximately 5 ​m above the ground. We combine both flight patterns, CCO facilitates for optimal 3D reconstruction of the plot center, while the grid pattern, ensures more uniform sampling across the plot. However, these approaches have inherent limitations in imaging the lower sections of the canopy, particularly as crops reach later developmental stages and the upper canopy becomes dense. While this is not a limitation we seek to address—given our focus on phenotyping panicle morphology—there are scalability concerns with existing methods. These studies rely on capturing still images at timed intervals while flying at very slow speeds, which restricts scalability and throughput. For example, Liu et al. [37] configure the UAV to fly at 0.2 ​m/s, capturing images every 2 ​s during a circular flight around a 5-m plot, resulting in a 5-min data collection period for 150 images of a single plot. Although this method minimizes motion blur and ensures sharp images, the operational cost in terms of battery usage and labour. We propose recording video while flying at higher speeds. The Ground Sampling Distance (GSD) for the images, based on the flight altitude and camera resolution, remains sufficiently high to support faster flight speeds. For the DJI Mavic 3E's sensor parameters, flying at 5 ​m results in a GSD of approximately 0.18 cm/pixel at 4K video resolution. The Mavic 3E can record video at 30 fps with a maximum shutter speed of 1/8000th of a second. Flying at 2.5 ​m/s will result in a motion blur of 0.3 ​mm, allowing for the imaging of a 6 ​m ​× ​6 ​m plot in approximately 20 ​s. This approach yields 600 frames (images) in the same duration, significantly reducing data acquisition time compared to still-image methods. Table 2 outlines our proposed protocol for the circular UAV flight plan. In addition to the circular flight we also flew the grid pattern flight, characterized by a cross-hatch trajectory with vertical and horizontal passes along the planting direction, with capture settings as outlined in Table 3.

**Table 2:** Proposed UAV acquisition plan - Circular Flights.

Flight Parameter	Setting
**Altitude**	5m
**Radius**	6m
**Capture**	Video (3840 ​× ​2160 pixels) 30fps @ 1/8000 ​s shutter speed
**Gimbal Angle**	45**^◦^**
**Flight Speed**	2.5 ​m/s
**Approximate Flight Duration Per Plot**	20 ​s
**Total images/frames per plot**	600

**Table 3:** Proposed UAV acquisition plan - Grid Flights.

Flight Parameter	Setting
**Altitude**	5m
**Image overlap across passes**	85 ​%
**Capture**	Video (3840 ​× ​2160 pixels) 30fps @ 1/8000 ​s shutter speed
**Gimbal Angle**	45**^◦^**
**Flight Speed**	4.5 ​m/s
**Approximate Flight Duration Per Plot**	30 ​s
**Total images/frames per plot**	900

**Fig. 1:**
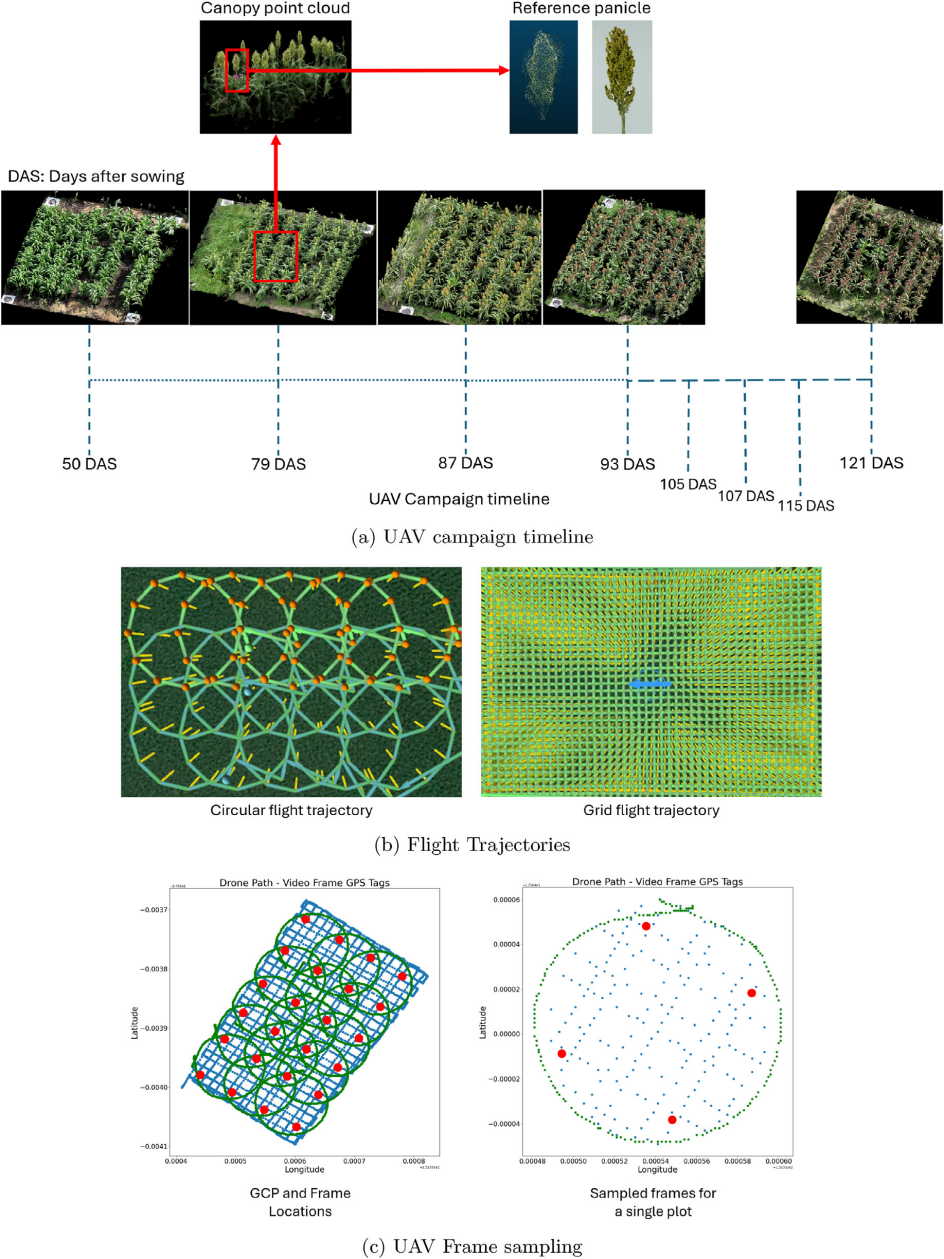
UAV campaign overview.

We used UGCS (https://www.sphengineering.com/flight-planning/ugcs) drone flight planning software to design and export flight paths for our UAV campaign. We made individual flight plans for plots grouped by treatment-repetition blocks, for both the circular and grid flights as described in Table 2, Table 3 Fig. 1b presents an overview of the flight trajectories over a single treatment-replication block, consisting of 15 plots. The UAV campaigns were conducted weekly from panicle emergence to crop maturity over an 8-week period, alternating between treatment blocks each week. For each plot, we randomly selected a reference panicle from the four central rows, tagging its peduncle with fluorescent pink tape to identify the panicle from the reconstructed 3D canopy model. Following the UAV campaigns, the reference panicles were harvested and transported to the lab, where they were imaged to generate high-quality 3D reference models. These reference models were used to assess and validate the accuracy of the UAV-based reconstructions, employing the methodology described by James et al. [53] for panicle 3D reconstruction. Fig. 1a provides an overview of the timeline for the UAV campaigns.

#### 3.1.3 UAV data processing

All UAV flights were conducted using a DJI Real Time Kinematic (RTK) base station to ensure precise positioning of the drone during data acquisition. In addition, four GCPs were placed at the corners of each plot during image collection. These GCPs acted as geo-referencing markers in the photogrammetry pipeline, ensuring high spatial accuracy and scale for both the mosaic and point cloud models. The GCP coordinates were measured using Emlid Reach RS2+ RTK Global Navigation Satellite System (GNSS) survey equipment. A Propeller Aeropoint was used to record the location of a reference GCP point, which was subsequently used to configure the Emlid Reach base station and record additional GCPs. For the entire trial, we collected a total of 88 GCP points. After capturing the canopy imagery using the drone, the video files undergo preprocessing to extract individual frames. The UAV's log associated with the video files is read to extract metadata from the Exchangeable Image File Format (EXIF) tags for each frame, including the GPS coordinates, camera angle and heading, and camera sensor details. For the 3D canopy reconstruction, each plot was processed separately, with a total of 400 frames sampled per plot—200 frames each from both the circular and grid flight paths. The frames were selected using the Farthest-Point Sampling (FPS) algorithm, which optimizes the distance between sampled frames to ensure maximum coverage. A 6m radius from the plot centre was used as a criterion to guide frame selection (based on the circular flight configuration). Fig. 1c shows the geolocations GCP locations (highlighted in red) and the geolocation of the recorded frames. It provides an overview of the GCP layout within a treatment-repetition block along with the frames sampled for the 3D reconstruction of an individual plot.

The captured images are imported into Agisoft Metashape [54] for photogrammetry, which applies an Sfm algorithm to stitch the images in 2D and then is used to generate a 3D point cloud model of the sorghum canopy, incorporating the GCP markers as references. These markers serve multiple purposes: they aid in georeferencing correction, act as tie points across the images for proper stitching, and function as scale bars to ensure the final reconstructed model is accurately scaled. To achieve this, the GCP markers must be manually identified and annotated in each image subset corresponding to the individual plots to correctly reference their imported coordinates. Moreover, the markers help identify plot corners and are used to sample frames for each plot, as mentioned previously. To streamline and automate the entire data processing workflow post-UAV flight, we employed fiducial markers proposed by Benligiray et al. [51] for GCP tagging. Specifically, we used STag markers due to their robust detection and localisation capabilities, even in scenes where partial occlusion occurs, such as in field trials where GCP markers may be obstructed by dirt, leaves, or other debris. After completing data collection, our automated point cloud reconstruction pipeline is as follows: first, the GCP detection step identifies and localizes all GCPs, then frames for individual plots are extracted. For each plot, the images, GCP detections, and georeference coordinates are processed using a Python script via the Agisoft Python Application Package Interface (API), which executes all photogrammetry steps and exports the final point cloud model (refer to Fig. 12 in supplementary materials section 7.2 to see camera alignment results). The input for this pipeline includes the raw video files, UAV logs, and a lookup table containing GCP IDs and plot corner coordinates (refer to Fig. 11 for GCP detection and localisation results in supplementary materials section 7.1).

#### 3.1.4 NeRF based point cloud reconstruction

**Fig. 2:**
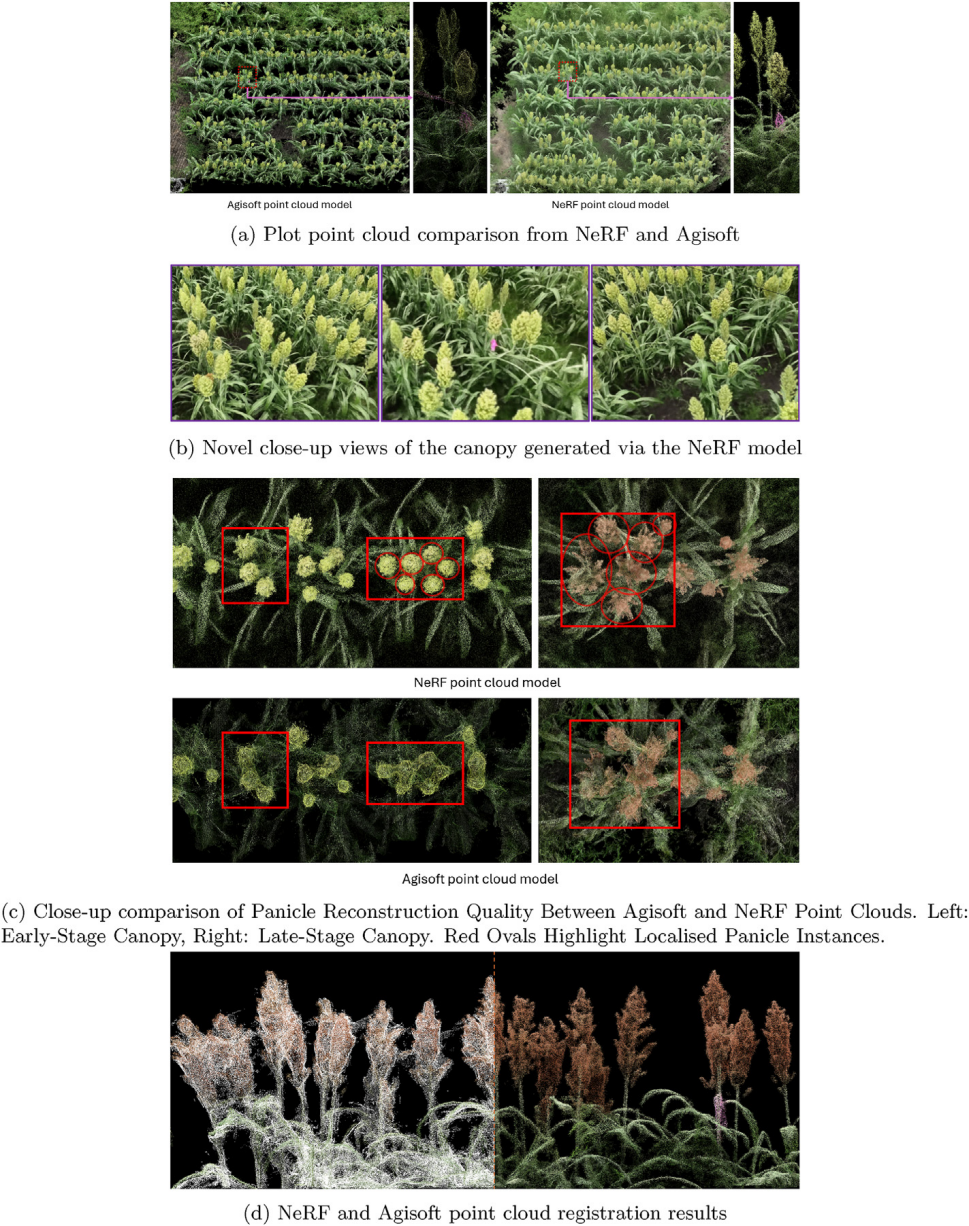
Point cloud models from Agisoft and NeRF models.

After the image alignment step in Agisoft, the estimated camera positions for each plot image set are exported to train a NeRFacto model using the NeRF Studio framework [28]. These images and their corresponding camera positions are then converted to the NeRF Studio format to create a dataset for model training. The primary objective of our study is the accurate 3D reconstruction of sorghum canopies. In this regard, we aligned our data collection criteria with the work by Arshad et al. [27], which evaluated several popular NeRF-based frameworks for 3D maize reconstruction using RGB video across multiple scales in real-world settings. Although our study involves a larger scale and region of interest (ROI) than those presented in Arshad et al.’s work, their results indicated that NeRFacto was the most accurate and robust model in terms of reconstruction precision and visual fidelity, outperforming other frameworks like Instant-NGP and TensorRF. As we prioritized reconstruction accuracy over computational efficiency in this step, we opted to train the largest variant of the NeRFacto model, ’NeRFacto-huge,’ which delivers the highest reconstruction quality among the NeRFacto models. The NeRF models were trained using the same image sets used in the Sfm-based photogrammetry pipeline in Agisoft, maintaining the native 4K resolution of the imagery. All models, including the photogrammetry pipeline, were trained on a system equipped with a single Nvidia RTX 4090 GPU with 24 ​GB of memory, 128 ​GB of RAM, and an Intel Core-i9 13900K processor. The approximate GPU memory consumption during NeRF model training was around 24 ​GB.When exporting the point cloud from the NeRF model, we crop the region of interest (ROI) to focus specifically on the area within each plot. The point cloud models generated by the NeRF models are then compared with those derived from the Sfm photogrammetry pipeline using Agisoft. The point cloud models from Agisoft contained approximately 35–45 million points per plot. To maintain consistency, we limited the export of point clouds from the NeRF models to 35 million points. A key issue observed during the visual inspection of the point cloud models from Agisoft was the presence of noise in high-density regions, particularly a webbing effect around panicles that were in close proximity or clustered together. This phenomenon complicates the identification and separation of individual panicles, consistent with the challenges reported by Chang et al. [39]. In contrast, when comparing these models with the point clouds derived from the NeRF model, we noted a significant improvement in quality. The NeRF-derived point clouds displayed greater clarity, and the panicle shapes were more easily distinguishable, facilitating the accurate localisation of individual panicles. Fig. 2 presents a comparison of the point cloud reconstruction results from Agisoft and the NeRF model, both derived from the same set of images. In Fig. 2a, the comparison highlights the canopy reconstruction of an entire plot. The Agisoft point cloud exhibits significant noise, making it difficult to discern the boundaries of individual panicles, whereas the NeRF point cloud offers more clearly defined panicle shapes and structures. Even leaf structures are more distinguishable in the NeRF point cloud, although both models struggle to capture individual leaf details in the lower canopy. Fig. 2b, shows close-up novel views of the canopy generated by the NeRF model. Finally, Fig. 2c compares close up Nadir views of an early-stage canopy point cloud model (same canopy model illustrated in Fig. 2a) and a late-stage canopy model. In the Agisoft point cloud model it is difficult to isolate single panicle instances within clustered panicles, while the enhanced reconstruction from the NeRF point cloud allows for a clear distinction among such panicles.

**Fig. 3:**
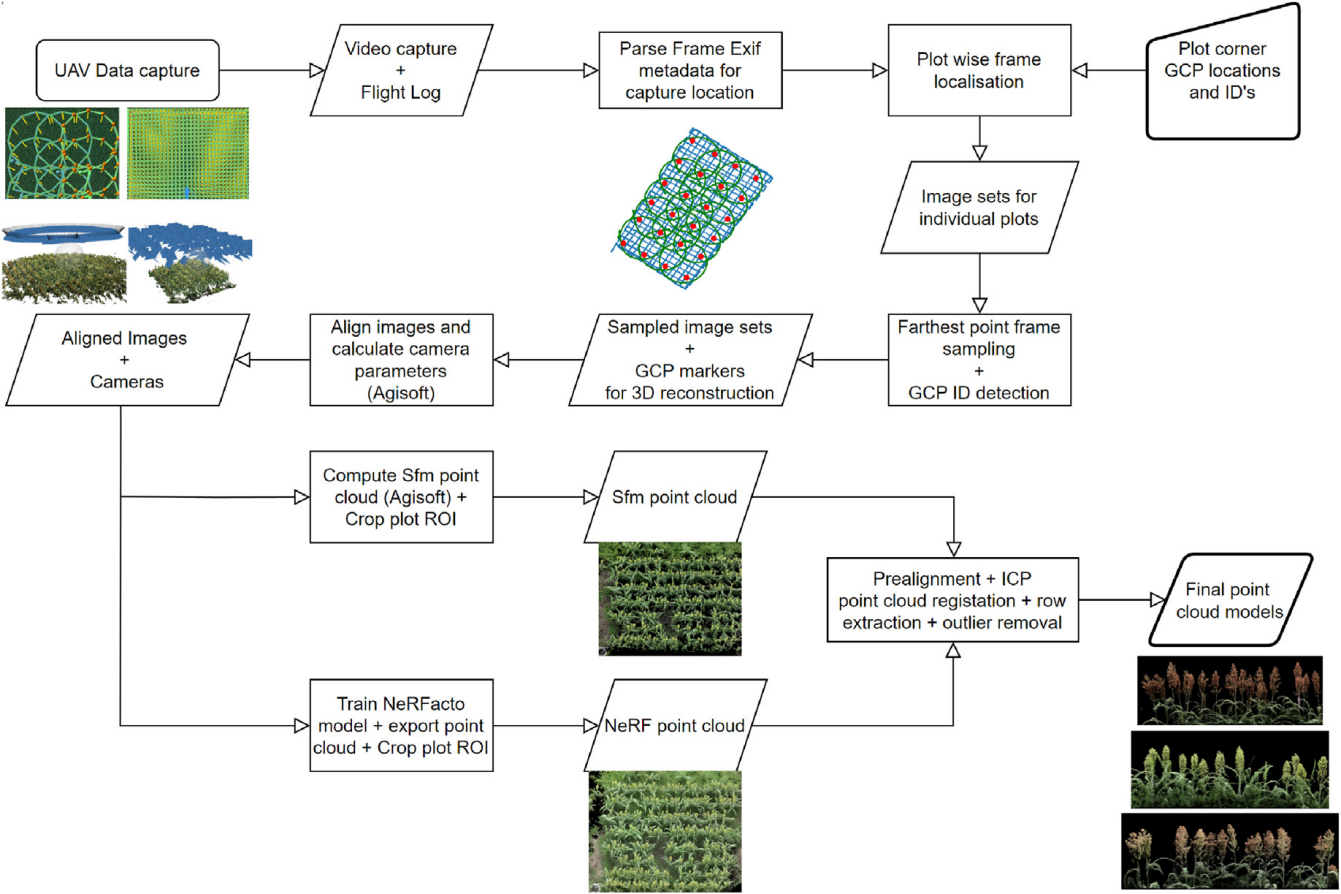
Pointcloud reconstruction flowchart.

To accurately retrieve the scale of the point cloud exported from the NeRF model, we perform co-registration with the point cloud generated by Agisoft. This is achieved using the Iterative Closest Point (ICP) algorithm, as implemented in CloudCompare [55], based on the method proposed by Besl et al. [56] and along with additions implemented by Masuda et al. [57]. Prior to applying the ICP, we preprocess the NeRF point cloud by scaling it to approximately match the scale of the georeferenced Agisoft point cloud. We then apply rotation and translation operations to align both point cloud centers to the origin. The ICP algorithm is applied iteratively, sampling 500,000 points per step, until the RMSE for the entire scene converges to 2 ​cm or less. During the registration, scaling, rotation, and translation adjustments are applied to the NeRF point cloud to achieve optimal alignment. Finally, after co-registering the NeRF point cloud to retrieve the correct scale, we proceed to crop and extract point cloud models for individual planting rows, followed by applying a denoising operation by removing outliers to clean up the point models to stage them for further analysis and processing. Fig. 2d shows the registration results for a single planting row, the left half shows the Agisoft point cloud overlayed (in white) on top of the registered NeRF point cloud and the right half displays the final cleaned point cloud model for the row, after outlier removal and noise reduction. Fig. 3 illustrates the complete overview of the point cloud reconstruction pipeline.

### 3.2 Synthetic 3D sorghum canopy generation model

#### 3.2.1 Generating individual plants

**Fig. 4:**
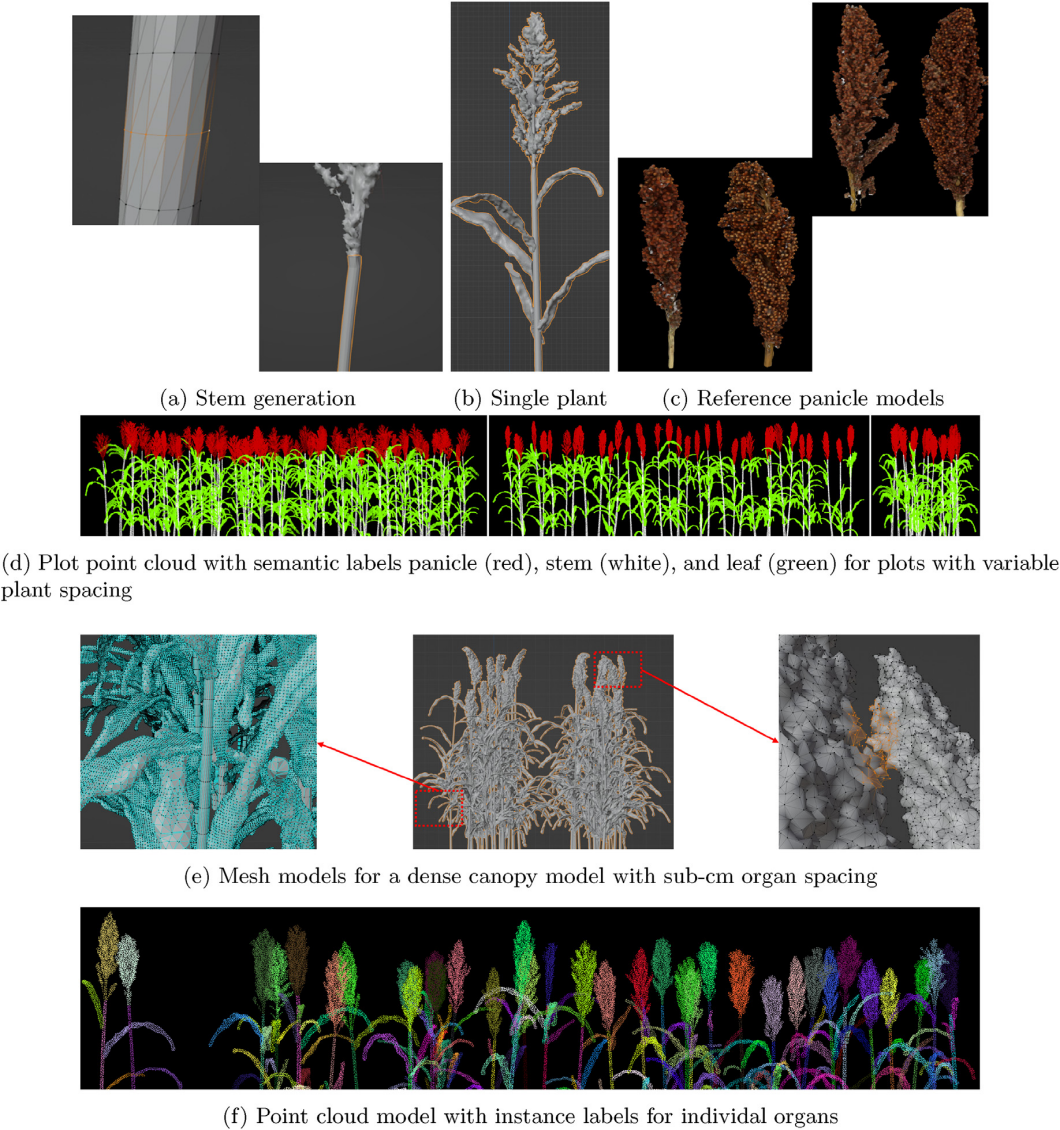
Annotated synthetic canopy models.

Our objective was to generate canopy architectures with an emphasis on using high-resolution and realistic panicle models. We developed a framework based on the dataset collected in our initial research chapter, which investigated the use of 3D panicle models for estimating grain count [53]. Additionally, we expanded our panicle model collection dataset by sampling and imaging panicle models from every plot in our experiment on a weekly basis after flowering. This allowed us to capture a range of panicle shapes, capturing both morphological variation due to genotypic differences and temporal changes in panicle structure throughout the grain-filling stage. To generate a single sorghum plant, we first interpolate a mesh model from a point cloud model of a sorghum panicle constructed using structure-from-motion imaging-based photogrammetry in Agisoft (Agisoft 2020). The geometry of the mesh model is pre-processed and cleaned in Blender [58] to eliminate overlapping edges, non-manifold geometry, and other errors, resulting in a watertight mesh model. The stem model for the plant is generated procedurally from the base of the panicle mesh model, which includes a part of the peduncle. We recursively fit cylinders to the base of the panicle to generate internodes, ensuring that the radius/thickness of the stem is consistent with the size of the peduncle. Additionally, the curvature and number of internodes of the stem can be specified during stem generation. We do not model the geometry of the roots, as our focus is solely on the above-ground architecture of the canopy. In our current framework iteration, we do not generate leaf geometry. Instead, we extract leaf models from real plant models provided by Gaillard et al. [19]. Although the plant models included in the dataset have instance segmentation results for the leaves from the skeletonization algorithm proposed by the authors, the segmentation results for the junction of the stem and leaf are too coarse to be extracted directly, so the leaf models were manually cleaned and pre-processed to obtain a set of mesh models for individual leaves. Subsequently, the leaf models are grafted onto the internode junctions of the stems to populate leaves on the sorghum plant. Fig. 4b shows the mesh model generated for a single plant.

#### 3.2.2 Generating plot models

To accurately construct canopy architectures that reflect real-world field conditions, we configure plant positioning and arrangement to align with the spatial configurations used in research and commercial plots. The generation of individual ”scenes” for each plot is done using a grid-based positioning scheme to place plants within a defined layout, implemented via Blender's Python API. Achieving high-quality annotated datasets for training deep learning models in tasks such as instance segmentation and object detection requires canopy structures with tightly packed and closely spaced components. It is therefore essential to ensure that the positioning of all geometries—stems, leaves, and panicles—within the generated canopy is consistent, without overlap, particularly in densely planted configurations. Our plot generation algorithm places plants sequentially in the grid, using Blender's implementation of the Bounding Volume Tree (BVH) structure proposed by Wal et al. [59]. Ray casting is employed to detect overlaps between the geometries, ensuring that there is no overlap between any of the components within the canopy model. To further enhance the realism of the generated canopy, we construct different ”plot types”, by sampling panicle models from a collection of morphologically grouped categories for each plot type, based on factors such as size, appearance, and developmental stage, allowing us to generate homogeneous plot configurations that still reflect natural variation (refer to Fig. 14 in supplementary materials section 7.4). For each plot type, we specify the plant spacing range and some additional parameters while generating the model for the plot. Algorithm 1 in the supplementary materials section 7.4 outlines the procedure for defining a grid-based plot layout and procedurally generating canopy architectures by populating the grid with panicle, leaf, and stem mesh models. Fig. 4 presents annotated 3D sorghum canopy models generated by our proposed algorithm. Fig. 4d displays point clouds of canopies with varying plant spacing and row length configurations, including semantic segmentation labels for the panicle, stem, and leaf components. Fig. 4e illustrates a mesh model of a dense canopy, where sub-centimeter spacing is maintained between individual plant organs. Fig. 4f Finally, shows instance labels for each component in a canopy.

### 3.3 SegVoteNet

SegVoteNet is proposed as a deep learning framework for semantic segmentation and 3D object detection on pure point cloud datasets. It utilizes segmentation results to generate localized object detection proposals, enhancing detection accuracy. Designed as a multi-task learning network, SegVoteNet integrates segmentation and detection within a dual-branch architecture, featuring a shared backbone.

**Fig. 5:**
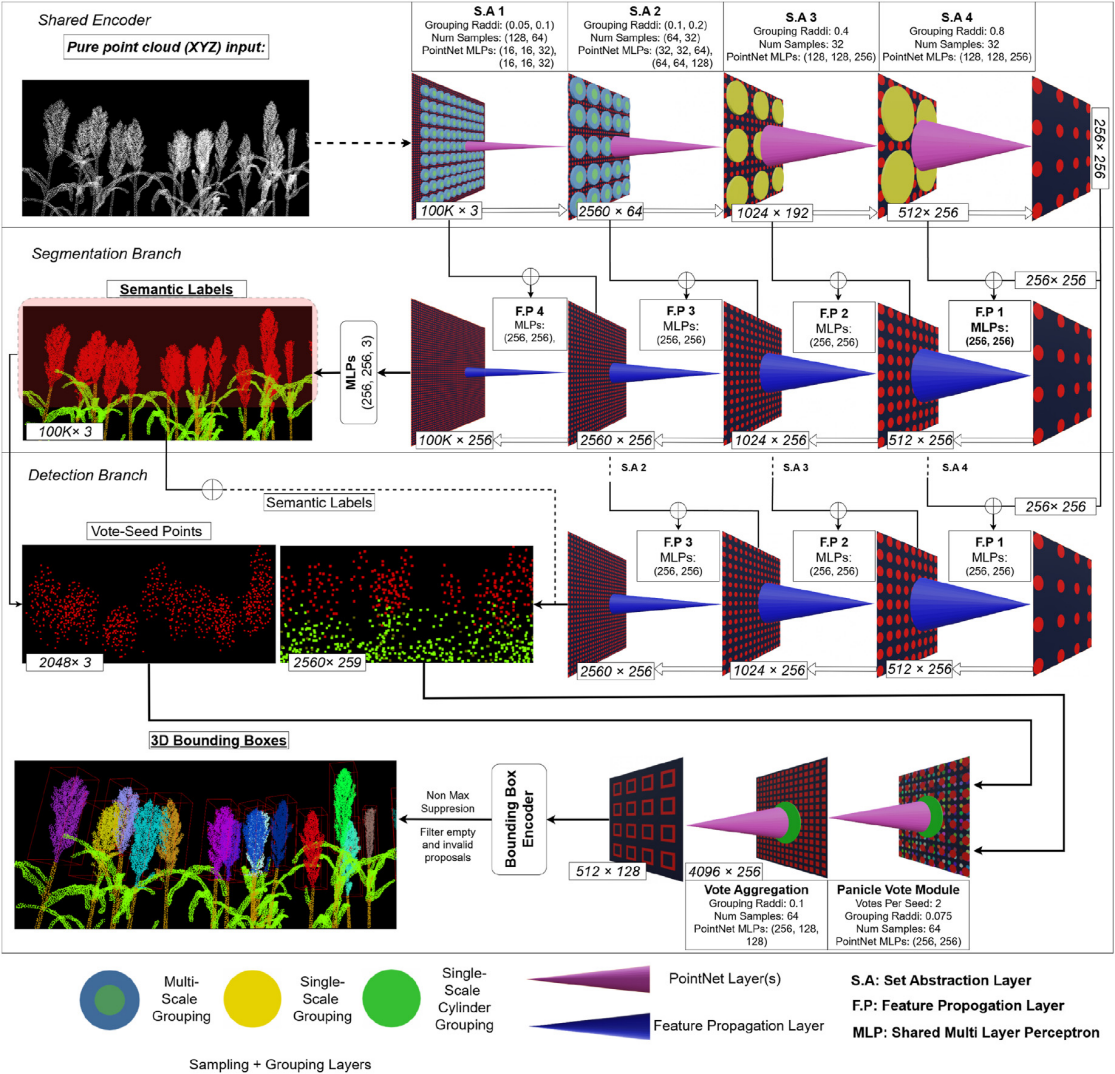
SegVoteNet architecture.

The architecture of SegvoteNet is derived from VoteNet [60] and PointNet++ [21], utilizing an encoder-decoder structure. The encoder comprises stacked Set Abstraction (SA) layers, each SA layer performs sampling and grouping operations to reduce the point set size. Specifically, the SA layer selects a subset from the original point set by sampling points based on FPS algorithm. For each selected point, it groups features from points lying within spherical ”receptive” fields, to consolidate local spatial relationships in an hierarchical learning fashion. These feature groups are processed through mini PointNet modules [61] to extract refined features for the reduced point set. With each subsequent SA layer in the encoder, the point sets are progressively downsampled, expanding the receptive field and increasing feature dimensionality. Conversely, the decoder uses Feature Propagation (FP) layers to upsample the point set back to its original size. The FP layers compute features for each point in the upsampled set by aggregating features from K-Nearest Neighbour (KNN) points, combining them via weighted averages based on inverse distances. The aggregated features are then concatenated with the original features of the corresponding point set from the respective SA layer in the encoder using skip connections. This merged feature set is further refined through a series of 1D convolutions. SegVoteNet is trained as a multi-task model for semantic segmentation and object detection, featuring a shared encoder with separate decoder branches for each task. We introduce several enhancements to the original VoteNet architecture within the detection branch. Firstly, we generate seed points for detection vote proposals by sampling only from points classified as ”panicle” by the segmentation branch, instead of relying directly on points from the final FP layer in the decoder. Moreover, rather than immediately applying MLPs to seed features for vote generation, we first apply a modified SA layer. This modified SA layer samples and groups points from the decoder based on the seed points from the segmentation branch, we label this section of the detection branch as ”Panicle Vote Module”. The following sections will detail the network's encoder, segmentation branch, and detection branch (including the panicle vote module). Fig. 5 outlines the architecture for SegVoteNet.

#### 3.3.1 Shared encoder

The segmentation and the detection branch have a shared encoder. The encoder is comprised of 4 SA layers (same as the original implementation of VoteNet), the encoder downsamples the original point cloud input progressively, while capturing features by aggregating local spatial relationships. We introduce a few modifications to the SA layers, firstly, the encoder uses a combination of Multi Scale Grouping (MSG) and Single Scale Grouping (SSG) SA layers instead of just SSG SA layers, as MSG layers were found to provide improved segmentation results for PointNet++. We also modify the sampling radii and sample size for the ball queries used in sampling and grouping operations for each of the SA layers based on the actual panicle dimensions. Additionally, we sample more points in the first SA layer, for covering the scene more densely. As planting density for in-field trials is often quite high, so we will have many dense and proximal instances of panicles across the scene to be detected. The first and second SA layers use MSG, while the third and fourth SA layers use SSG, additionally each SA layer uses the XYZ-coordinates for each point by concatenating them to the learned feature vectors. The first SA layer samples the original input point cloud to 2560 points and applies 2 ball queries of radii 0.05 and 0.1 for grouping points, samples 128 and 64 points for each query respectively, the grouped features from the are processed through PointNets with MLPs of size [16,16,32]. The second SA layer samples 1024 points and applies 2 ball queries of radii 0.1 and 0.2, samples 64 and 32 points for each query respectively, the grouped features from the are processed through PointNets with MLPs of size [32,32,64] and [64, 64, 128]. The third SA layer samples 512 points, applies a single ball query with radii 0.4, samples 32 points, and processes the features through a PointNet with [128, 128, 256] MLP, and the fourth SA has the same configuration as the third layer, but uses a ball query with an increased radius of 0.8. The final output is a downsampled point cloud with 256 points, with a 256-length feature vector. All point cloud dimensions are specified in metres, and the ball query radii for SA layers were chosen based on dimensions of the panicle models we used in our 3D canopy generation model (Section 3.2).

#### 3.3.2 Segmentation branch

The segmentation branch consists of four FP layers, each equipped with shared MLP layers of size [256, 256]. These FP layers iteratively upsample the point cloud back to its original size, followed by a final shared MLP layer of size [256, 256, 3], which generates logits for segmentation. The segmentation branch is trained to classify each point into three classes: panicle, stem, or leaf. To address class imbalance within the canopy model, where leaf points are predominant and stem points are sparse, we apply a weighted cross-entropy loss with class weights of 0.3 for panicle, 0.6 for stem, and 0.1 for leaf. This weighting emphasizes on accurate classification of stem and panicle points while reducing the impact of the leaf-dominant point distribution.

#### 3.3.3 Detection branch

##### 3.3.3.1 Seed point sampling

**Fig. 6:**
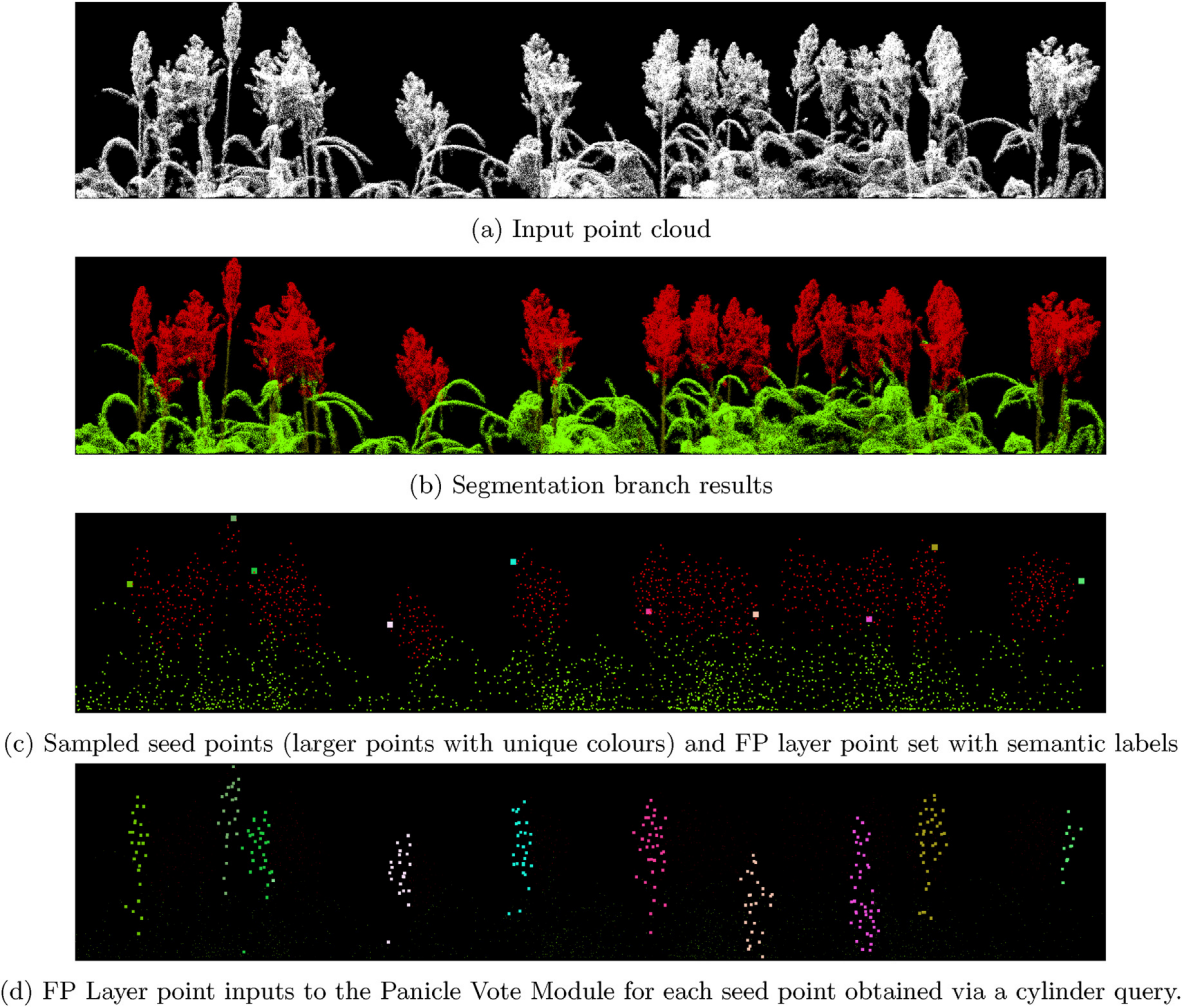
Seed point sampling and Panicle Vote Module input.

In VoteNet's original implementation, seed points for vote generation—used in creating object proposals—were sampled from points in the final FP layer. Since each SA layer applies a series of FPS operations, the coverage of the point set is evenly distributed across the entire scene, suitable for general detection tasks involving randomly distributed multi-class objects. However, in panicle detection, most panicles are concentrated in the canopy's upper region and are densely packed. The vote generation module, which proposes object centers and features for bounding box parameters, relies on the partial or complete coverage of the object of interest within the receptive field of the seed point to generate reliable proposals for detection. When using FPS-based sampling, seed points will include areas like leaf edges or lower canopy sections, disconnected structurally from the panicles, leading to less reliable object proposals. To address this, our approach uses segmentation results to sample seed points more effectively: we apply FPS only on points identified as panicles. This approach directs the model's focus to the relevant canopy regions, producing more reliable object detection votes. Furthermore, by only sampling across panicle points the models can generate proposals suitable for detecting closely spaced panicle instances within dense canopies. Fig. 6c shows sampled seed points from points classified as panicles.

##### 3.3.3.2 Cylinder grouping

The SA layers and vote aggregation module in VoteNet traditionally use ball queries to sample points within spherical receptive fields, facilitating the learning and aggregation of local and global features. For our detection branch, we propose a modification: using cylindrical sampling queries instead of spherical ball queries for generating vote proposals and vote aggregation step. Specifically, we sample points within a circular base of a radius equivalent to a ball query, extending vertically to encompass the canopy's entire height along the z-axis. Since seed points for vote proposals are already localised in panicle regions, a cylindrical sampling and grouping approach enables us to include associated stem and leaf points within the receptive field, thus generating more contextualized votes. Furthermore, this cylindrical kernel aligns better with the elongated structure of panicles, thus offering a more effective receptive field. Fig. 6d shows points sampled and grouped from the detection branch's FP layer point set via applying cylinder queries to seed points.

##### 3.3.3.3 Panicle vote module

In the original implementation of VoteNet, the point set and features from the final SA layer are used to generate votes for object proposals. This voting module calculates object center offsets (vote points) and vote features (used to estimate bounding box dimensions, orientation, and class) by passing the seed points and their features through a shared MLP layer, which is subsequently processed through an additional SA module to aggregate votes for the final object proposals. In our proposed ’Panicle Vote Module,’ we introduce an additional SA step to refine vote points and vote features. As described, seed points are selected from the subset of original points classified as panicle points in the segmentation branch. Seed features for each seed point are generated by applying a SA operation over the point set and features from the last FP layer, using a cylinder query with a base radius of 0.075 to sample and group features around each seed point. Additionally, semantic labels are concatenated as one-hot encoded vectors to feature vectors for all points in the final FP point set. The aggregated features are then processed through a PointNet using [256, 256] MLPs, producing seed features for each point, which serve as inputs to the standard voting module (a shared MLP of [256, 256]). This module processes the seed points and features to generate vote offsets and vote features, producing two votes per seed point. The vote points and features then pass through a vote aggregation module, which applies another SA operation with a cylinder query with a base radius of 0.1 and a PointNet with [256, 128, 128] MLPs to aggregate votes and features. Finally, the aggregated vote features pass through shared MLP layers to predict bounding box classifications and regression scores for estimating box dimensions, orientation, and calculating losses. The detection branch is trained using the loss functions proposed in the original VoteNet paper. Fig. 6 shows the sampling results for seed points and their respective aggregated point set inputs for the panicle vote module from the final FP layer.

### 3.4 Dataset and model training

SegVoteNet was trained on a synthetic point cloud dataset of sorghum canopies, as described in section 3.2. For training, we cropped each plot model to include only the upper half of the canopy, sampling 100,000 points using FPS to ensure balanced spatial distribution. The training dataset consisted of 600 synthetic plot models with plant spacing configurations varying between 5 and 15 ​cm, along with broadly homogenous collections of panicle shapes and sizes, with specific variations as described in section 3.2.2. Each plot model was constructed to be approximately 4 ​m of a single row. To introduce additional variability, some plots were randomly modified to contain small contiguous areas without plants or panicles. The training data included semantic segmentation labels, categorizing the point cloud into panicle, leaf, and stem classes as specified in section 3.3.2. In addition to, 3D bounding box annotations for each panicle instance, described by the location and box dimensions for the XYZ axis, and box rotation and orientation with respect to the x-axis.

During training, we applied standard point cloud augmentation techniques, including rotation, point dropout, noise addition, point shuffling, and rescaling. Rotation was constrained to the xy-plane, and point dropout was applied with probabilities uniformly sampled between 0.2 and 0.5 for each sample. Noise was introduced on a class-wise basis, with leaf points receiving more translation than panicle and stem points to preserve the canopy structure. Additionally, while applying noise we ensured that translated points did not merge with panicle points to maintain consistent detection and segmentation labels in augmented samples. The point cloud models were rescaled with scaling factors sampled randomly between 0.85 and 1.15 to preserve the general dimensions of the panicles, as the detection branch categorizes panicles into four distinct size classes based on their average height, width, and length dimensions of the bounding boxes, the panicles were grouped into their respective size classes according to their rough morphological characteristics.

#### 3.4.1 Model implementation

SegVoteNet was implemented using the MMDetection3D toolbox [62], a Python-based deep learning framework built on PyTorch [63] (Model checkpoints and data set samples will be available on GitHub (https://www.github.com/chr15-j/SegVoteNet)). The model was trained over 10,000 epochs with a batch size of 8, consistent with the original VoteNet experiments on the SUN RGB-D and ScanNetV2 datasets. We employed the AdamW optimizer [64] with a multi-step learning rate scheduler. The learning rate, initialized at 0.002, decays by a factor of 0.1 ​at 24 and 32 epochs, mirroring MMDetection3D's VoteNet implementation for SUN RGB-D and ScanNetV2, adjusted here for the reduced batch size (from 32 to 8). The segmentation branch uses a weighted cross-entropy loss, as detailed in section 3.3.2. The detection branch is trained on the losses as described in the original VoteNet paper, as implemented within the MMDetection3D framework, which includes vote loss and objectness loss for proposals generated by the voting module. Bounding box estimation losses include class and residual losses for both size and orientation, along with the bounding box centre loss. Fig. 13 in the supplementary section 7.3 presents the training loss log for the model.

## 4 Results

### 4.1 Panicle reconstruction results

The reference panicles from each plot were harvested and imaged in the lab for 3D reconstruction, creating high-resolution ground truth models for assessing panicle reconstruction accuracy. Although the harvested panicles were transported and imaged on the same day as the UAV data collection, visual inspection and comparison of the field and lab point cloud models revealed some structural changes in the panicles during the time between harvesting and imaging. These structural changes varied by panicle type: closed and compact panicles largely retained their original structure, whereas open and loose panicles showed more pronounced alterations (flatttening or change in shape during transport).

**Fig. 7:**
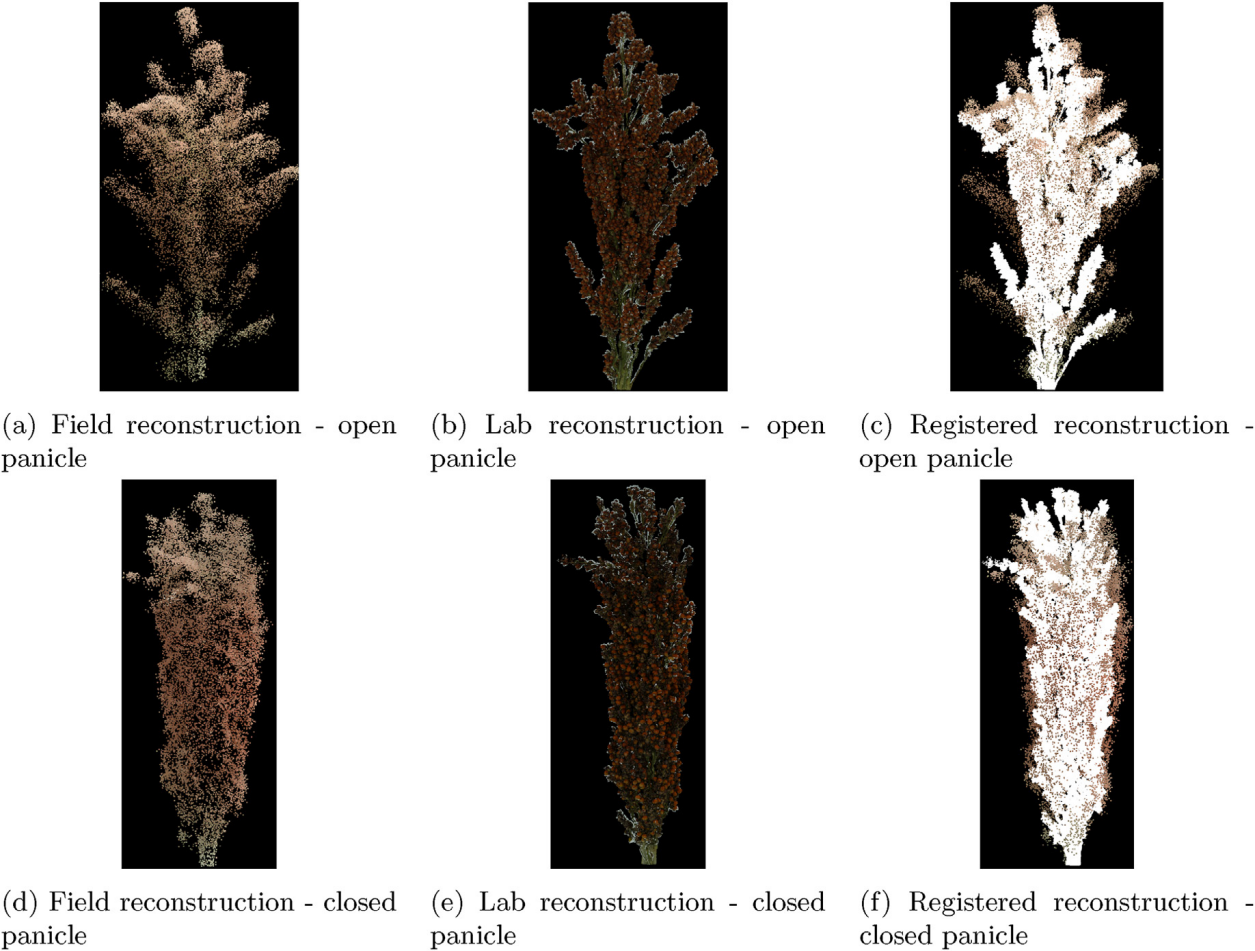
Field vs Lab Panicle reconstruction.

Fig. 7 compares two pairs of panicle reconstructions between the UAV flight and the lab. Fig. 7c and f presents the registered point cloud models, with the lab reconstructions shown in white and the field reconstructions depicted as colored point clouds. Fig. 7a and d displays the field reconstructions, while Fig. 7b and e shows the lab reconstructions. The point clouds were registered using ICP, aligning them through translation and rotation while preserving the original scale of the models to validate reconstruction quality.

The open panicle in Fig. 7c shows a good overall alignment of the panicle shape but reveals several shifted branches that fail to align precisely. Conversely, the closed panicle in Fig. 7f demonstrates a much closer alignment between the field and lab reconstructions, with minimal displacement of branches. Overall, the panicles were observed to contract after harvesting (likely partly due to water loss), leading to a slight reduction in both their overall length and width.

After co-registering the panicle reconstructions from the lab and field, 3D bounding boxes were fitted to calculate the length, width, and height of the panicles. Table 4 reports the Pearson correlation coefficient, MAE, and RMSE for panicle dimension estimates obtained from the field and lab reconstructions. This evaluation was conducted on 32 panicles, consisting of four samples collected weekly from the experiment after panicle emergence.

**Table 4:** Estimated panicle dimensions.

Correlation/Error	Panicle length	Panicle Width	Panicle height
**r**	0.938	0.894	0.984
**MAE(cm)**	1.182	1.781	0.322
**RMSE(cm)**	1.557	2.363	0.438

### 4.2 SegVoteNet

#### 4.2.1 Validation set metrics and model generalisation

During training, the model was evaluated on the validation set at every 10-epoch interval. We had 2 validation sets to assess model performance and generalisation ability. The first validation set comprised 70 synthetic plot models with detection, and segmentation labels for all three classes (synthetic validation dataset). The second validation set included 10 real plot point cloud models collected from our experiment at various growth stages (weekly samples from panicle emergence to maturity) across different varieties. This real validation dataset was manually annotated with 3D bounding box labels using the Segments.ai annotation tool (https://segments.ai/), containing 343 individual panicle instances in total. To ensure a comprehensive evaluation, validation samples were carefully selected to include challenging scenarios, such as small and developing panicles at earlier growth stages, as well as dense canopies containing open and proximal panicles. For this dataset, segmentation evaluation was restricted to panicle class points, as the dataset lacked explicit semantic segmentation labels, and the panicle segmentation labels were interpolated from the 3D bounding box annotations.

For detection validation metrics, we evaluate the mAP at both 0.25 and 0.5 IOU thresholds across the synthetic and real validation sets. For segmentation, the synthetic validation set is assessed using overall average class accuracy, while the real validation set is evaluated based on panicle segmentation accuracy only. During inference, rather than including all non-empty bounding box proposals in the Non Max Suppression (NMS) step, we apply a filtering process. We check for point occupancy within the central 20 ​% of the volume by center cropping the bounding box proposals along the xy plane. Followed by, excluding any bounding boxes that lack points classified as panicles. Given that point cloud density is fairly uniform across the scene, we do not need to account for sparse panicle instances with empty centers.

**Fig. 8:**
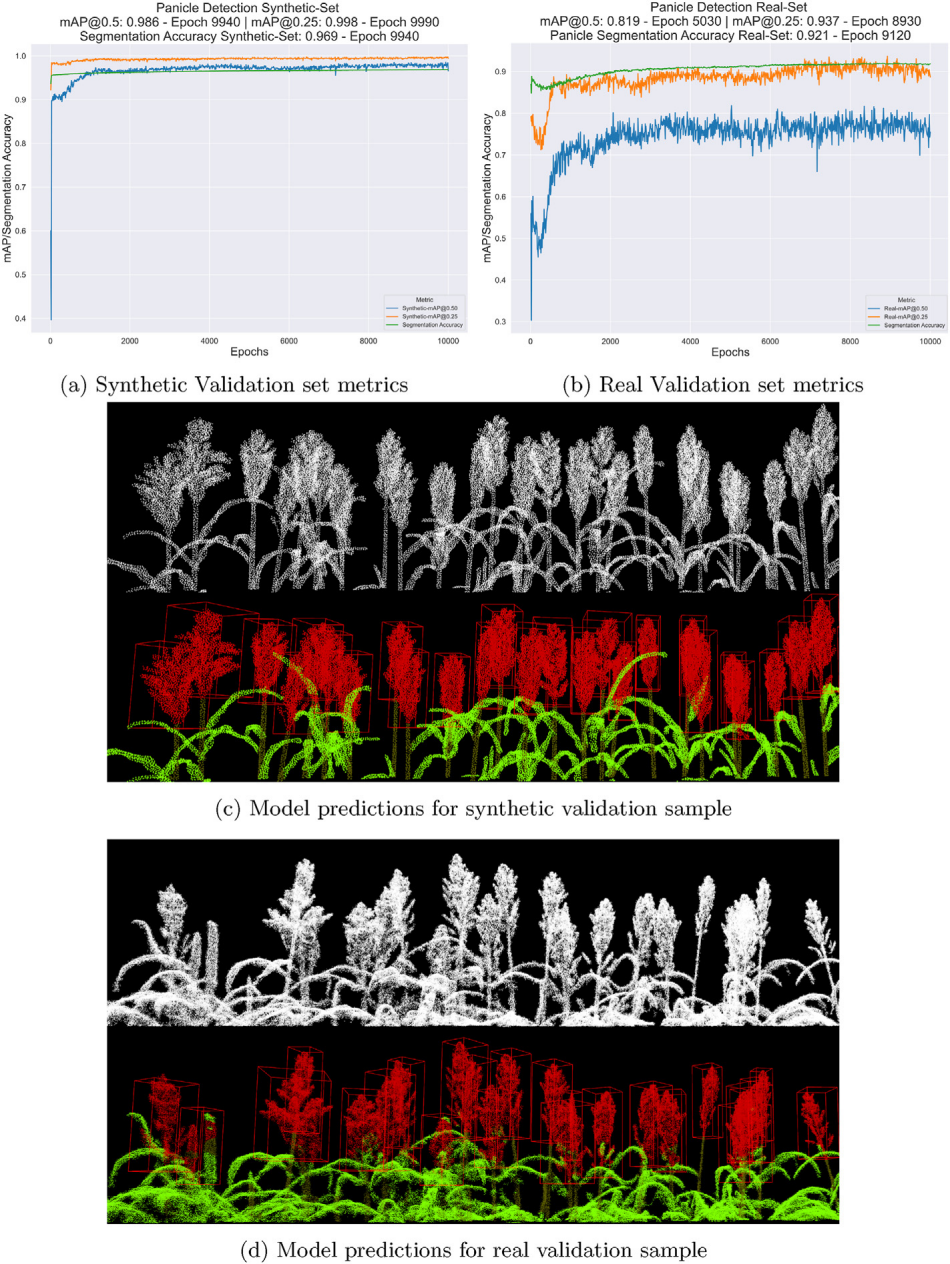
Synthetic and real validation set.

Fig. 8 illustrates the performance metrics for the model across validation sets during training. In Fig. 8a, the detection and segmentation metrics for the synthetic validation set demonstrate robust performance, with the model achieving a high detection accuracy of 0.986 mAP at 0.5 IOU and 0.998 mAP at 0.25 IOU, alongside an overall segmentation accuracy of 0.969 across all three classes. Additionally, the validation metrics show rapid improvement and convergence. Fig. 8b shows the performance on the real validation set, the model attains lower performance metrics (given the domain gap across the datasets), achieving 0.819 mAP at 0.5 IOU and 0.937 mAP at 0.25 IOU for panicle detection, with a panicle segmentation accuracy of 0.921. Fig. 8 also shows sample point clouds along with model detection and segmentation results from the synthetic validation set (Fig. 8c) and real validation set (Fig. 8d).

#### 4.2.2 Test set performance

The model checkpoints, selected based on their performance on the synthetic and real validation sets, were further evaluated on a separate test set. This test set consisted of 40 manually annotated single-row point cloud models, derived from five plot-level point cloud models corresponding to five randomly selected plots across the experimental setup. These samples spanned eight weeks of growth after panicle emergence, as detailed in Section 3.1.2, and included 1337 individual panicle instances. The model selected on the real validation set achieved a detection performance of 0.850 mAP @ 0.5IOU, while the model selected on the synthetic validation set achieved a comparable performance of 0.814 mAP @ 0.5IOU. Table 5 summarizes the detection performance metrics, including mAP, Mean Average Recall (mAR), and panicle segmentation accuracy.

**Table 5:** Model test set performance.

Model Selection	Synthetic Validation Set	Real Validation Set
**mAP@0.25IOU**	**0.945**	0.934
**mAR@0.25IOU**	0.965	0.965
**mAP@0.50IOU**	0.814	**0.850**
**mAR@0.50IOU**	0.896	**0.918**
**Panicle Segmentation**	0.934	**0.936**

**Fig. 9:**
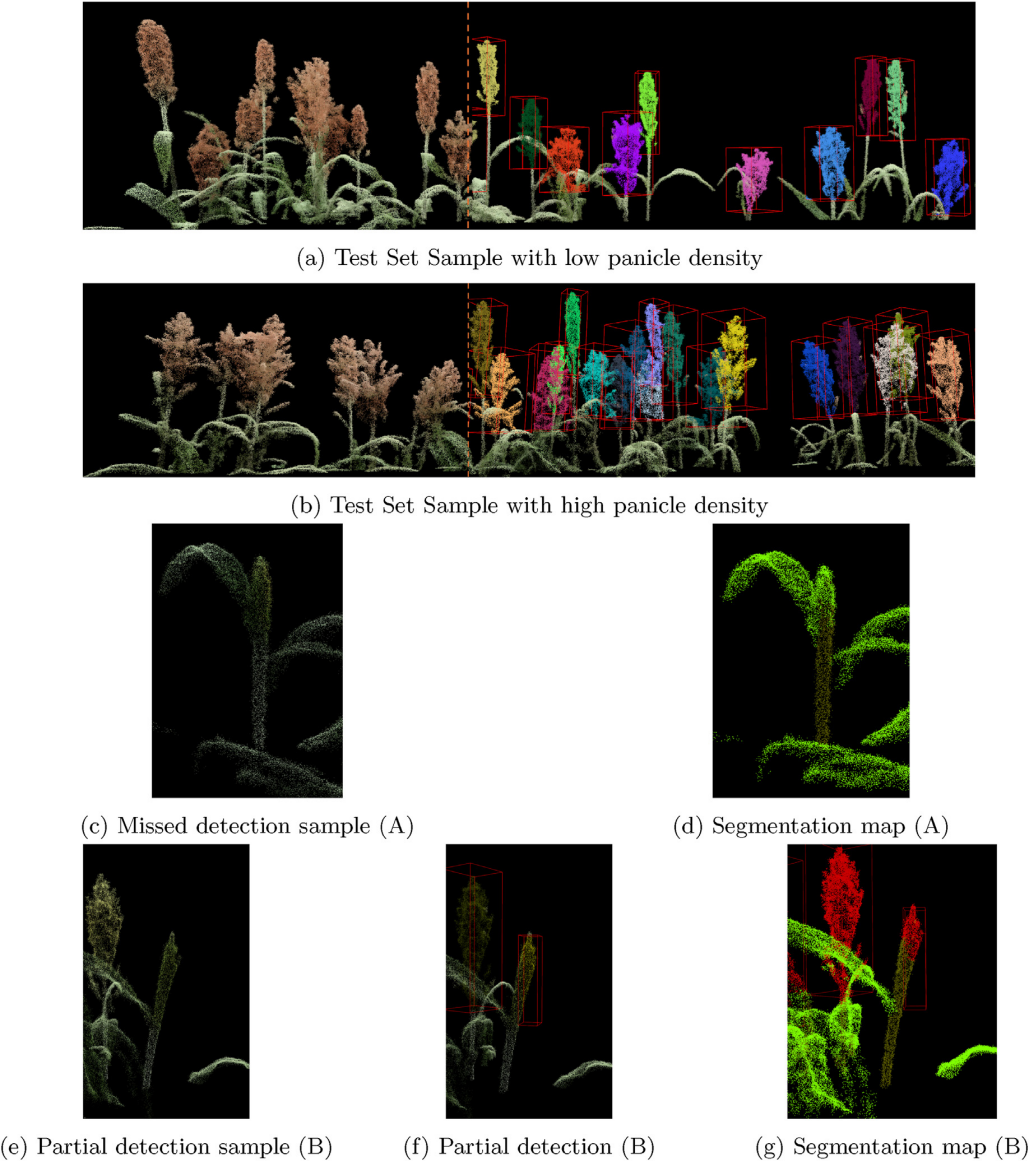
Test set inference.

Fig. 9 illustrates the model's detection results on selected test set samples. Specifically, Fig. 9a highlights the model's predictions for a plot characterized by low panicle density and wide spacing between panicles. Fig. 9b depicts predictions for a dense canopy with closely packed panicles.

Upon examining the model predictions on the test set, it was observed that the model struggles to detect small, emerging panicle instances, e.g. when the panicle is partially sheathed within the flag leaf (detecting panicle right after the panicle initiation stage), for which there are no cases in the synthetic training set. Fig. 9 illustrates two examples of these challenges, in Fig. 9c and d, the point cloud and segmentation map show a complete panicle misclassification, with most points labeled as stem and the rest as leaf. Fig. 9e shows partial detection, where the detection output (Fig. 9f) is incomplete, and the segmentation map (Fig. 9g) misclassifies parts of the panicle as stem.

#### 4.2.3 Ablation experiments

To evaluate the efficacy of our proposed modifications and model architecture, including the Panicle Vote Module, we undertook a comparative analysis of detection performance across several models. This analysis included several variations of VoteNet, starting with the original model trained solely with detection labels, alongside versions incorporating our proposed modifications and modules progressively, as described below:

• **VoteNet trained with only detection labels**, utilizing the same backbone as SegVoteNet and employing a 0.3-radius ball query for the vote aggregation module, consistent with the original implementation.
• **VoteNet with segmentation branch**, featuring the same shared backbone architecture as SegVoteNet.
• **VoteNet with segmentation branch ​+ ​semantic labels** concatenated to the seed point features.
• **SegVoteNet without cylinder grouping operation.**
• **SegVoteNet with cylinder grouping operation.**

All models were trained and validated on the same dataset, adhering to identical batch sizes and training configurations as detailed in section 3.4. We compare the model training loss for the detection branch across all models, along with the mAP evaluated at 0.25 and 0.5 IOU thresholds for the synthetic and real validation sets. Table 6 summarizes these metrics across all models, with the highest-performing value for each metric highlighted in bold. Table 7 compares test set performance across all models.

**Table 6:** Ablation study - model performance comparison on real and synthetic validation sets.

Model	Detection Loss *↓*	Synthetic Val set mAP *↑*		Real Val set mAP *↑*	
		@0.25IOU	@0.5IOU	@0.25IOU	@0.5IOU
VoteNet -	0.664	0.896	0.747	0.868	0.634
VoteNet ​+ ​Segmentation Branch	0.676	0.886	0.655	0.891	0.620
VoteNet ​+ ​Segmentation Branch ​+ ​Seed class labels	0.702	0.900	0.744	0.874	0.623
SegVoteNet without Cylinder Grouping	0.399	0.995	0.965	0.922	0.751
SegVoteNet ​+ ​Cylinder grouping	**0.383**	**0.998**	**0.986**	**0.937**	**0.819**

**Table 7:** Ablation study - model performance comparison on real test set.

Model	Real Test set			
	mAP@ 0.25IOU *↑*	mAR@ 0.25IOU *↑*	mAP@ 0.5IOU *↑*	mAR@ 0.5IOU *↑*
VoteNet -	0.903	0.936	0.679	0.811
VoteNet ​+ ​Segmentation Branch	0.893	0.943	0.625	0.788
VoteNet ​+ ​Segmentation Branch ​+ ​Seed class labels	0.868	0.940	0.670	0.826
SegVoteNet without Cylinder Grouping	0.933	0.961	0.812	0.892
SegVoteNet ​+ ​Cylinder grouping	**0.934**	**0.965**	**0.850**	**0.918**

## 5 Discussion

### 5.1 3D reconstruction and synthetic canopy generation model

Our 3D reconstruction pipeline demonstrated consistent and reliable results from a video-based data acquisition which shortens capture times and enhances scalability. Across different growth stages, the dimensions of reconstructed panicles from field data and from reference 3D models imaged in the lab were highly correlated, especially for panicle height (length). The pipeline prioritizes the quality of canopy reconstruction using offsite computational resource constraints. Future work should explore more efficient NeRF-based approaches, such as 3DGS-based methods [36], which may enable near real-time 3D reconstruction for field-based phenotyping applications.

The synthetic sorghum canopy generation model extends the utility of manually scanned 3D reconstructions of panicle and leaf models. Our approach focuses on procedurally generating full canopy models with consistent geometry and organ spacing to generate realistic field scenarios. The morphological diversity in our synthetic canopy models is currently constrained by diversity of scanned 3D panicle and leaf models (especially for emerging panicles, see section 4.2.2). To address these constraints, future work couldutilise a fully procedural generation model using advanced geometric algorithms or generative 3D deep learning approaches, such as PolyGen [65] or SDM-Net [66]. Such models permit generation of synthetic outputs with greater morphological variety as would inclusion of more diverse observed data. SegVoteNet was trained exclusively on pure point cloud models with the goal of testing its performance on NeRF and Sfm-derived datasets. Integrating the synthetic sorghum canopy generation model with LiDAR simulation frameworks like Helios++ [67] could facilitate the generation of point clouds tailored to LiDAR-based data acquisition methods, which are becoming increasing available in portable instruments. Incorporating specialized data augmentation techniques suitable for LiDAR point clouds could further aid in developing robust panicle detection and canopy segmentation methods optimised for LiDAR-derived datasets. Since SegVoteNet operates without relying on color or normals, it is directly applicable to high-resolution LiDAR data and we propose to test it as such in future.

### 5.2 SegVoteNet: 3D-Object detection for phenotyping panicles from point clouds

We propose SegVoteNet as a multi-task learning framework that optimizes detection performance by leveraging the relationships across segmentation and detection tasks. The training loss log and validation set metrics underscore its efficacy, with steady loss decline and convergence observed in both the detection and segmentation branches during training. On the synthetic validation set, SegVoteNet achieves remarkably high detection accuracy (0.986 mAP@0.5IOU), converging rapidly to comparable performance within 30 epochs (around 0.90 mAP@0.5 IOU). the model also demonstrated robustness and generalisability when evaluated on an additional validation dataset comprised of manually annotated point cloud models for real sorghum canopies (0.819 mAP @ 0.5IOU).

While the model exhibited slower convergence on the real cf synthetic validation sets it improved steadily over epochs, maintaining consistency with the training loss log trends. The results from the ablation study further validate the efficacy of the SegVoteNet framework and its components. SegVoteNet was superior to the original VoteNet on synthetic (0.986 vs 0.747 mAP @ 0.5IOU) and real validation datasets (0.819 vs 0.634 mAP @ 0.5IOU). Notably, the study highlights the effectiveness of the Panicle Vote Module, cf variants of VoteNet trained with an additional segmentation branch which were poorer or comparable in detection to vanilla VoteNet. This reinforces the validity of the multi-task learning design of SegVoteNet. VoteNet and PointNet++ are well suited as foundation models for SegVoteNet as they are seminal deep learning models for processing point clouds natively for detection and segmentation tasks. Future work to improve adaptability will explore more recent variants of point feature extraction modules, specifically based on graph and transformer architectures.

We compared SegVoteNet's performance to existing methods for sorghum panicle detection and counting (Table 8) and find that it generally improves upon or delivers comparable results. Notably, most existing methods rely on RGB images with more uniform panciles compared to ours which vary substantially in size, as well as in the shapes, sizes, colors, and developmental stages of panicles. For example, when using 3D panicle detection methods [22,39] tested against a much narrower range of panicle measurements (length and width). Considering only test sets focusing on single or dual-planted rows, similar to our samples and compared to image-based approaches, SegVoteNet achieves comparable object detection performance (mAP) and generally better counting accuracy (MAE, *R*^2^, MAE%). The exception is RetinaNet [68] in Ghosal et al. [10], which reports superior detection performance (on a more uniform dataset which we originally collected), although SegVoteNet exhibits lower counting error. Additionally, the CNN-based crowd-counting approach from Oh et al. [69] achieves a lower MAE. For 3D point cloud-based methods, SegVoteNet offers improved counting performance and more accurate estimation of panicle dimensions.

**Table 8:** Sorghum panicle count phenotyping methods.

Deployment	Data	Methodology	Metrics v/s SegVoteNet	Reference
**Unmanned Aerial Vehicle (UAV)**	**RGB Image**	Decision tree-based image segmentation ​+ ​visual bag of words	*R*^2^: 0.88 — **0.951**	(Guo et al., 2016) [70]
		RetinaNet	*R*^2^: 0.88 — **0.951** mAP@0.5: **0.94** — 0.85	(Ghosal et al., 2019) [10]
		SegNet ​+ ​connected components	MAE: 1.88~2.66 — **1.28**	(Malambo et al., 2019) [71]
		2-stage CNN for density map estimation	MAE: **1.06** — 1.28	(Oh et al., 2019) [69]
		UNet ​+ ​contour detection	MAE%: 5 ​% — **4.3** ​%	(Lin and Guo 2020) [72]
		RetinaNet, YoloV5	MAE: 1.5 — **1.28** mAP@0.5: **0.862** — 0.85	(Cai et al., 2021) [73]
		Pix2Pix based synthetic training data generation ​+ ​Yolov5	MAE: 4.6 — **1.28** mAP@0.5: 0.79 — **0.85** MAE%: 9.7 ​% — **4.3** ​%	(Cai et al., 2022) [74]
	**Point Cloud** ​+ ​**Colors**	Colour Thresholding ​+ ​shape fitting	*R*^2^: 0.83 — **0.951** Height r: 0.62 — **0.984** Width r: 0.83 — **0.894**	(Chang et al., 2021) [39]
**Terrestrial LiDAR Scanner (TLS)**		Colour thresholding Density-based&clustering	*R*^2^: 0.921 — **0.951** MAE: 3.1 — **1.28** MAE%: 9.97 ​% — **4.3** ​% Height r: 0.88 — **0.984** Width r: 0.79 — **0.894**	(Malambo et al., 2019) [22] *Results interpolatedfrom graphs/figures in paper*

**Fig. 10:**
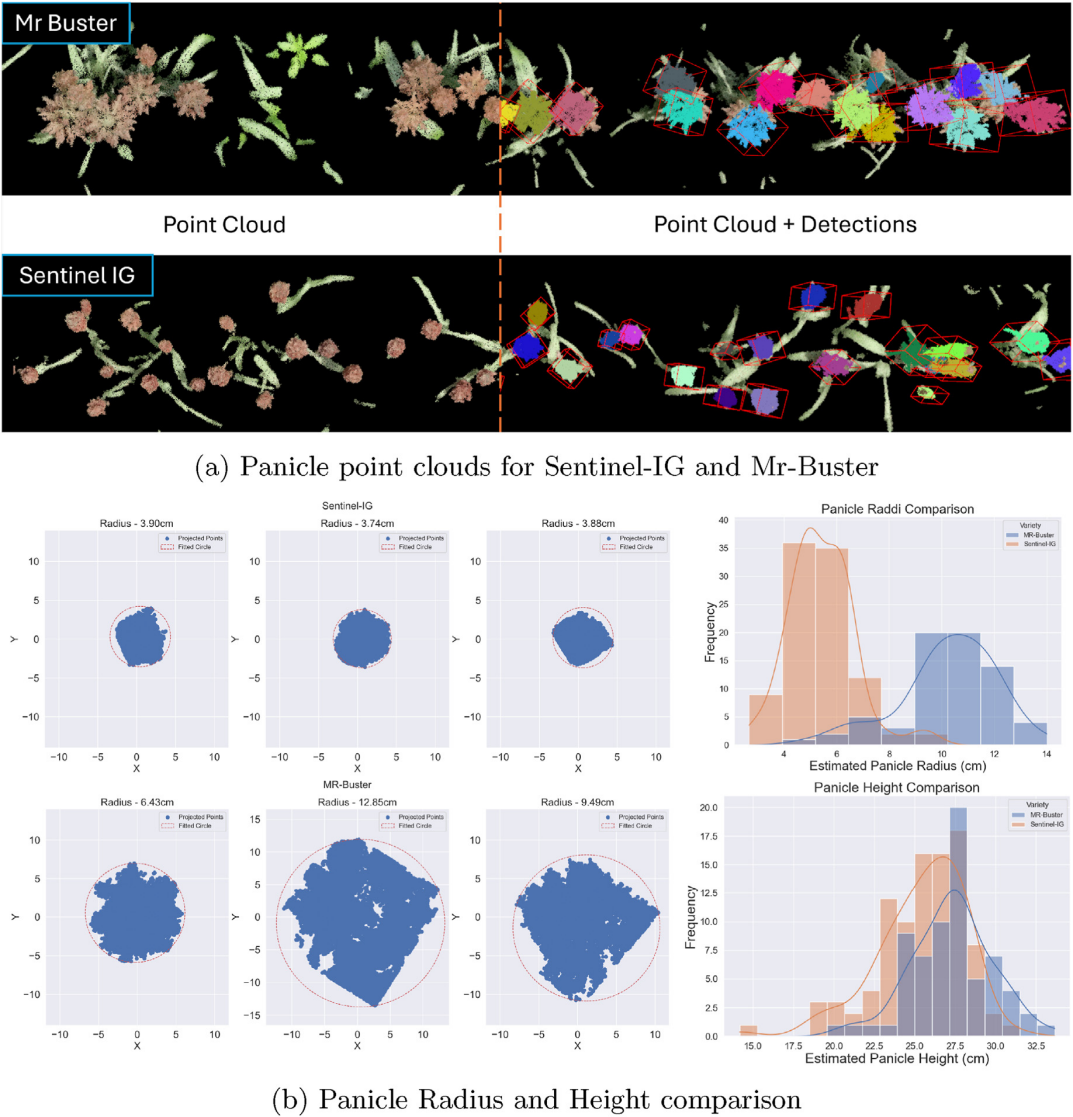
Comparing panicle morphology across different varieties.

The results from SegVoteNet can be integrated into phenotyping workflows for both breeding experiments and commercial trials. In large-scale commercial trials, SegVoteNet can contribute to yield prediction tools by combining morphological traits such as panicle count and total panicle volume with variety-specific prior data on grain yield, including average grain size and grain weight. However, given the logistical challenges of UAV-based data acquisition for every plot in extensive trials, targeted sampling can be implemented. Subsections of trials may be selected based on variation observed through high-altitude UAV flights, leveraging information such as Digital Elevation Models and Vegetation Indices. This approach enables the development of calibration models linking low-altitude 3D reconstruction flights with scalable high-altitude orthomosaic mapping. For breeding trials, SegVoteNet facilitates tracking panicle morphology over time, classifying panicle architecture, and comparing structural differences across genotypes. For example, Fig. 10 illustrates a comparison of panicle size metrics (panicle radius and panicle height) derived from SegVoteNet's 3D detection results for two sorghum hybrids, Mr-Buster and Sentinel-IG, from the rain-fed treatment blocks. Mr-Buster exhibits an open-panicle type, while Sentinel-IG has a semi-open panicle (Fig. 10a). Although both varieties share similar panicle height distributions, Mr-Buster was found to have more than double the panicle radius of Sentinel-IG, albeit with fewer panicle numbers. Additionally, the panicle radius histogram reveals a clear distinction in panicle architecture profiles between the two hybrids (Fig. 10b). Future research will focus on integrating panicle geometry extracted from 3D object detection with high-resolution RGB imagery to develop methods for estimating grain count per panicle and analyzing variation within plots and across genotypes [53]. Furthermore, beyond extracting panicle-related traits, 3D point cloud models can also be used to monitor canopy architecture changes over time, estimate traits like biomass and leaf area index, and quantify growth dynamics.

The proposed data acquisition protocol and the SegVoteNet architecture are well suited for adaptation to 3D panicle or head detection tasks in other cereal crops (maize, wheat, barley, rice and millet), and tasks such as counting bolls in cotton. The method should suit detection of dense and closely spaced organs in the upper canopy, whenever the morphology of the detected organ is resolvable (and even if the colour or texture may be similar to the canopy itself). UAV data acquisition must be carefully planned to minimise the effects of wind on the canopy, and extending the method requires the development of synthetic data generation frameworks to minimise labelling effort when developing new scalable phenotyping systems.

## 6 Conclusion

This study presents a comprehensive framework for phenotyping 3D sorghum panicle morphology in field trials. We propose a scalable approach for large-scale 3D reconstruction of sorghum canopies using drone-based RGB imagery. This method integrates traditional Sfm-based photogrammetry with NeRFs to achieve enhanced and accurate 3D canopy reconstructions. Additionally, we introduce SegVoteNet, a multi-task deep learning framework designed for 3D object detection and semantic segmentation. SegVoteNet incorporates a novel object proposal module that leverages segmentation outputs to optimize detection proposals. The model was trained exclusively on procedurally generated pure point cloud datasets of synthetic sorghum canopy models. It was evaluated on synthetic and real-world datasets to test its generalization capabilities. SegVoteNet demonstrated very high detection performance on synthetic validation datasets, achieving 0.986 mAP @ 0.5IOU, and importantly, maintained robust detection accuracy on real validation and test datasets, achieving 0.819 mAP @ 0.5IOU and 0.85 mAP @ 0.5IOU without fine-tuning or additional training. However, the model faces challenges in reliably identifying small, emerging panicles that remain partially sheathed within the flag leaf, as this specific scenario was not represented in the synthetic training dataset.

## Author contributions

“C. James: Conceptualization, Methodology, Data curation, Software, Formal analysis, Investigation, Writing - original draft, Visualisation.”

“S. S. Chandra: Conceptualization, Methodology, Supervision, Writing - review and editing, Project administration.”

“S. C. Chapman: Conceptualization, Methodology, Supervision, Writing - review and editing, Resources, Project Administration, Funding acquisition.”

## Data availability

The datasets and models used in the findings of this study are available on GitHub (https://github.com/chr15-j/SegVoteNet).

## Funding

This project was funded in part by the 10.13039/501100000980Grains Research and Development Corporation (10.13039/501100000980GRDC) of Australia UOQ2003-011RTX ‘Innovations in plant testing in Australia’. Additionally, we acknowledge the use of the facilities, and scientific and technical assistance of the Australian Plant Phenomics 10.13039/100031212Network (APPN), which is supported by the Australian Government's National Collaborative Research 10.13039/100031425Infrastructure Strategy (NCRIS). Chrisbin James was a recipient of The University of Queensland's Research Training Program scholarship and a top-up scholarship from GRDC UOQ2306-005RSX.

## Declaration of competing interest

The authors declare that they have no known competing financial interests or personal relationships that could have appeared to influence the work reported in this paper.
