# Supplementary material for: A scalable and efficient UAV-based pipeline and deep learning framework for phenotyping sorghum panicle morphology from point clouds
Source: Plant Phenomics. 2025 May 19;7(2):100050. doi: 10.1016/j.plaphe.2025.100050 (PMC12709908; doi:10.1016/j.plaphe.2025.100050)
Supplement: Multimedia component 1 [file mmc1.pdf]

Supplementary Materials

GCP Detection

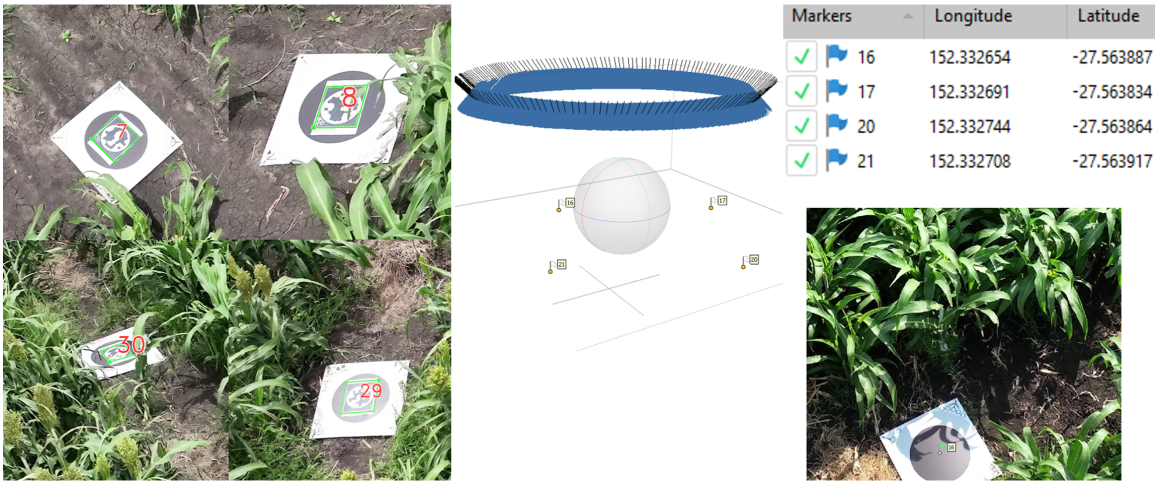

Figure 1: Ground Control Point (GCP) detection for processing images

Example of Camera Alignment Results from Agisoft Metashape

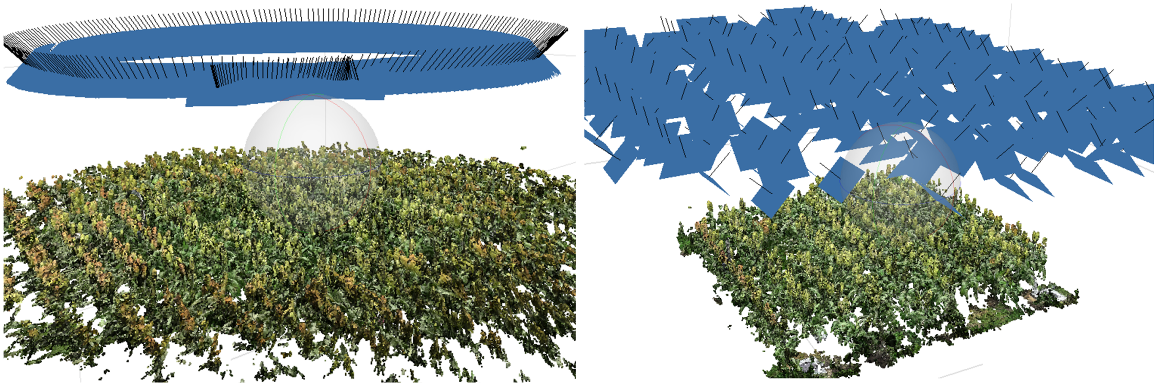

Figure 2: Estimated camera positions for a single plot: Circular flight left, grid flight right

Training Loss Log

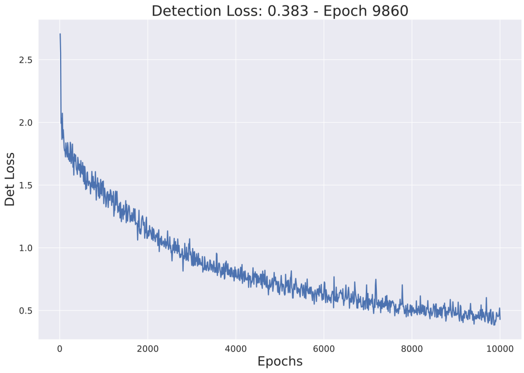

(a) Detection branch loss

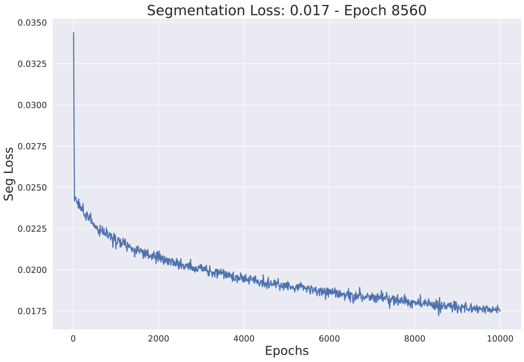

(b) Segmentation branch loss

Figure 4: SegVoteNet - Model Training log

Plot Generation Algorithm based on Mesh Models for Panicle, Leaf and Stem Models

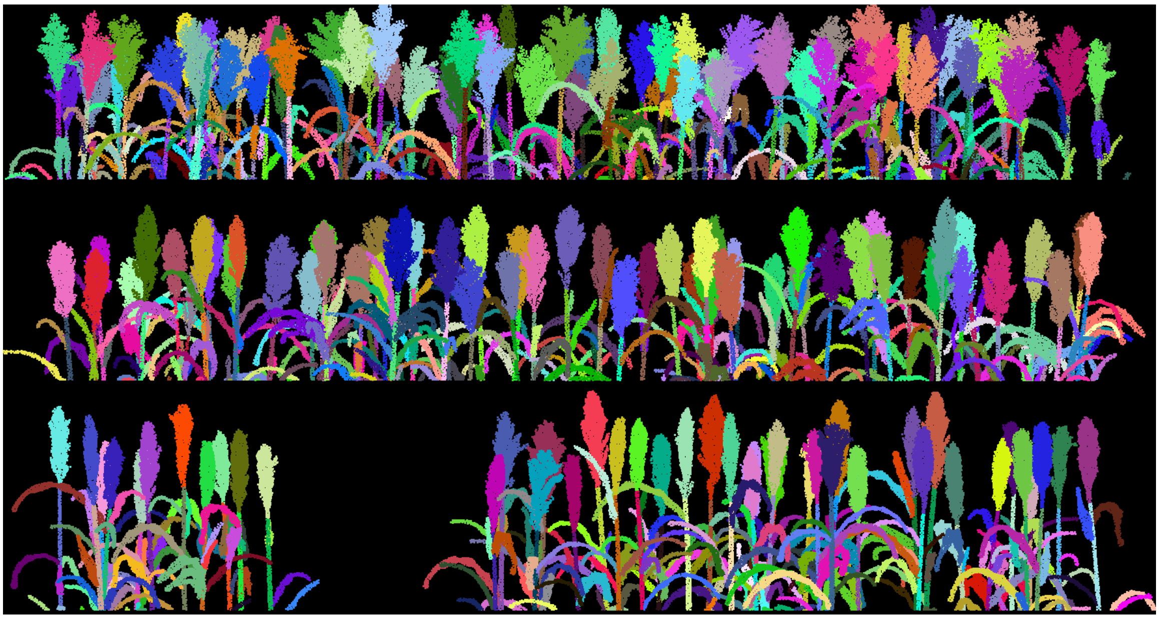

Figure 5: Examples of Plots generated with Open, Semi-Open, and Thin Panicle Models

```
Input: Number of plots n_plots, plot types plot_types, plot length
       plot_length, spacing distribution spacing_distribution, plant
       leaf range (leaves_min, leaves_max), grid deviation (X_DEV,
       Y_DEV, Z_DEV), lodging angles (X_ROT, Y_ROT, Z_ROT)
for plot_idx = 1 to n_plots do
    Set plot type plot_type = plot_types[plot_idx%len(plot_types)];
    Set plant spacing plant_spacing = spacing_distribution[plot_type];
    number of plants N_PLANTS = round(plot_length/plant_spacing);
    for row = 1 to N_ROWS do
        Generate grid coordinates for each plant in row GRID;
        Set random deviations for each plant position:
          GRID[row, plant_num] += uniform sample ((0, X_DEV),
          (0, Y_DEV), (0, Z_DEV));
        Set rotation for each plant: Plant_lodge[row, plant_num] =
          uniform sample ((0, X_ROT), (0, Y_ROT), (0, Z_ROT));
    end for
    Initialize plot_mesh as None;
    Generate randomised mesh collection for plot type panicle_list;
    for row = 1 to N_ROWS do
        for plant_num = 1 to N_PLANTS do
            Load panicle and stem models panicle_mesh, stem_mesh;
            Set and apply rotations to panicle and stem
              (Plant_lodge[row, plant_num]);
            Translate models to grid location (GRID[row, plant_num]);
            Set collision collision = check collision (plot_mesh,
              (panicle_mesh, stem_mesh));
            while collision ≠ 0 do
                Calculate vector between closest intersecting vertices and
                  translate panicle_mesh and leaf_mesh ;
                Set collision = check collision (plot_mesh,
                  (panicle_mesh, stem_mesh));
            end while
            add panicle_mesh and stem_mesh to plot_mesh;
            Get available internode junctions on stem mesh
              avail_internodes;
            for leaf_num = 1 to uniform
              sample(leaves_min, leaves_max) do
                for leaf_tries = 0 to max_leaf_tries do
                    Load leaf model from randomized mesh collection
                      leaf_mesh;
                    Set attached = False;
                    for internode in avail_internodes do
                        Translate leaf_mesh to internode;
                        if check collision (plot_mesh, leaf_mesh) == 0
                          then
                            Translate leaf_mesh to internode;
                            Update avail_internodes;
                            Set attached = True;
                            add leaf_mesh to plot_mesh ; break;
                        end if
                    end for
                    if attached then
                        break;
                    end if
                    delete leaf_mesh
                end for
            end for
        end for
    end for
end for
```

Algorithm 1: Plot Generation algorithm
